# Rituximab versus tocilizumab in anti-TNF inadequate responder patients with rheumatoid arthritis (R4RA): 16-week outcomes of a stratified, biopsy-driven, multicentre, open-label, phase 4 randomised controlled trial

**DOI:** 10.1016/S0140-6736(20)32341-2

**Published:** 2021-01-23

**Authors:** Frances Humby, Patrick Durez, Maya H Buch, Myles J Lewis, Hasan Rizvi, Felice Rivellese, Alessandra Nerviani, Giovanni Giorli, Arti Mahto, Carlomaurizio Montecucco, Bernard Lauwerys, Nora Ng, Pauline Ho, Michele Bombardieri, Vasco C Romão, Patrick Verschueren, Stephen Kelly, Pier Paolo Sainaghi, Nagui Gendi, Bhaskar Dasgupta, Alberto Cauli, Piero Reynolds, Juan D Cañete, Robert Moots, Peter C Taylor, Christopher J Edwards, John Isaacs, Peter Sasieni, Ernest Choy, Costantino Pitzalis, R4RA collaborative group, Charlotte Thompson, Serena Bugatti, Mattia Bellan, Mattia Congia, Christopher Holroyd, Arthur Pratt, João Eurico Cabral da Fonseca, Laura White, Louise Warren, Joanna Peel, Rebecca Hands, Liliane Fossati-Jimack, Gaye Hadfield, Georgina Thorborn, Julio Ramirez, Raquel Celis

**Affiliations:** a Centre for Experimental Medicine and Rheumatology, Queen Mary University of London, London, UK; b Institute of Health Sciences Education, Queen Mary University of London, London, UK; c Department of Rheumatology, Mile End Hospital, Barts Health NHS Trust, London, UK; d Department of Rheumatology, Cliniques Universitaires Saint-Luc, Brussels, Belgium; e Institute of Experimental and Clinical Research, Université catholique de Louvain, Brussels, Belgium; f Centre for Musculoskeletal Research, Division of Musculoskeletal & Dermatological Sciences, The University of Manchester, Manchester, UK; g National Institute for Health Research (NIHR) Manchester Biomedical Research Centre, Manchester, UK; h Department of Cellular Pathology, Barts Health NHS Trust, London, UK; i Department of Rheumatology, Kings College Hospital NHS Foundation Trust, London, UK; j Department of Rheumatology, Fondazione IRCCS Policlinico San Matteo, University of Pavia, Pavia, Italy; k Rheumatology Department, Guy's and St Thomas' NHS Foundation Trust, London, UK; l The Kellgren Centre for Rheumatology, Manchester Royal Infirmary, Manchester University NHS Foundation Trust, Manchester, UK; m Rheumatology Department, Hospital De Santa Maria, Centro Hospitalar Universitário Lisboa Norte, Lisbon, Portugal; n Rheumatology Research Unit, Instituto de Medicina Molecular João Lobo Antunes, Faculdade de Medicina, Universidade de Lisboa, Lisbon, Portugal; o Skeletal Biology and Engineering Research Centre, Department of Development and Regeneration, Katholieke Universiteit Leuven, Leuven, Belgium; p Division of Rheumatology, University Hospital Leuven, Leuven, Belgium; q Department of Rheumatology, University of Eastern Piedmont and Maggiore della Carita Hospital, Novara, Italy; r Rheumatology Department, Basildon Hospital, Basildon, UK; s Rheumatology Department, Mid & South Essex University Hospitals NHS Foundation Trust, Southend University Hospital, Westcliff-on-Sea, UK; t Rheumatology Unit, Department of Medicine and Public Health, Azienda Ospedaliero-Universitaria and University of Cagliari, Monserrato, Italy; u Department of Rheumatology, Homerton University Hospital, London, UK; v Rheumatology Department, Hospital Clínic de Barcelona, Barcelona, Spain; w Institut d'Investigacions Biomèdiques August Pí I Sunyer, Barcelona, Spain; x Academic Rheumatology Unit, Aintree University Hospital, Liverpool, UK; y Faculty of Health, Social Care and Medicine, Edge Hill University, Ormskirk, UK; z Nuffield Department of Orthopaedics, Rheumatology and Musculoskeletal Sciences, Botnar Research Centre, University of Oxford, Oxford, UK; aa NIHR Clinical Research Facility, University Hospital Southampton, Southampton, UK; ab Faculty of Medicine, University of Southampton, Southampton, UK; ac Translational and Clinical Research Institute, Newcastle University, Newcastle upon Tyne, UK; ad Musculoskeletal Unit, Newcastle upon Tyne hospitals NHS Foundation Trust, Newcastle upon Tyne, UK; ae King's Clinical Trials Unit, Kings College London, London, UK; af CREATE Centre, Cardiff University, Cardiff, UK; ag Department of Rheumatology, University Hospital of Wales, Cardiff, UK

## Abstract

**Background:**

Although targeted biological treatments have transformed the outlook for patients with rheumatoid arthritis, 40% of patients show poor clinical response, which is mechanistically still unexplained. Because more than 50% of patients with rheumatoid arthritis have low or absent CD20 B cells—the target for rituximab—in the main disease tissue (joint synovium), we hypothesised that, in these patients, the IL-6 receptor inhibitor tocilizumab would be more effective. The aim of this trial was to compare the effect of tocilizumab with rituximab in patients with rheumatoid arthritis who had an inadequate response to anti-tumour necrosis factor (TNF) stratified for synovial B-cell status.

**Methods:**

This study was a 48-week, biopsy-driven, multicentre, open-label, phase 4 randomised controlled trial (rituximab *vs* tocilizumab in anti-TNF inadequate responder patients with rheumatoid arthritis; R4RA) done in 19 centres across five European countries (the UK, Belgium, Italy, Portugal, and Spain). Patients aged 18 years or older who fulfilled the 2010 American College of Rheumatology and European League Against Rheumatism classification criteria for rheumatoid arthritis and were eligible for treatment with rituximab therapy according to UK National Institute for Health and Care Excellence guidelines were eligible for inclusion in the trial. To inform balanced stratification, following a baseline synovial biopsy, patients were classified histologically as B-cell poor or rich. Patients were then randomly assigned (1:1) centrally in block sizes of six and four to receive two 1000 mg rituximab infusions at an interval of 2 weeks (rituximab group) or 8 mg/kg tocilizumab infusions at 4-week intervals (tocilizumab group). To enhance the accuracy of the stratification of B-cell poor and B-cell rich patients, baseline synovial biopsies from all participants were subjected to RNA sequencing and reclassified by B-cell molecular signature. The study was powered to test the superiority of tocilizumab over rituximab in the B-cell poor population at 16 weeks. The primary endpoint was defined as a 50% improvement in Clinical Disease Activity Index (CDAI50%) from baseline. The trial is registered on the ISRCTN database, ISRCTN97443826, and EudraCT, 2012-002535-28.

**Findings:**

Between Feb 28, 2013, and Jan 17, 2019, 164 patients were classified histologically and were randomly assigned to the rituximab group (83 [51%]) or the tocilizumab group (81 [49%]). In patients histologically classified as B-cell poor, there was no statistically significant difference in CDAI50% between the rituximab group (17 [45%] of 38 patients) and the tocilizumab group (23 [56%] of 41 patients; difference 11% [95% CI −11 to 33], p=0·31). However, in the synovial biopsies classified as B-cell poor with RNA sequencing the tocilizumab group had a significantly higher response rate compared with the rituximab group for CDAI50% (rituximab group 12 [36%] of 33 patients *vs* tocilizumab group 20 [63%] of 32 patients; difference 26% [2 to 50], p=0·035). Occurrence of adverse events (rituximab group 76 [70%] of 108 patients *vs* tocilizumab group 94 [80%] of 117 patients; difference 10% [–1 to 21) and serious adverse events (rituximab group 8 [7%] of 108 *vs* tocilizumab group 12 [10%] of 117; difference 3% [–5 to 10]) were not significantly different between treatment groups.

**Interpretation:**

The results suggest that RNA sequencing-based stratification of rheumatoid arthritis synovial tissue showed stronger associations with clinical responses compared with histopathological classification. Additionally, for patients with low or absent B-cell lineage expression signature in synovial tissue tocilizumab is more effective than rituximab. Replication of the results and validation of the RNA sequencing-based classification in independent cohorts is required before making treatment recommendations for clinical practice.

**Funding:**

Efficacy and Mechanism Evaluation programme from the UK National Institute for Health Research.

**Research in context**

**Evidence before this study**

We searched PubMed for clinical trials, observational studies, and review articles with the search terms “rheumatoid arthritis”, “rituximab”, “B cells” or “B lymphocytes”, and “synovial membrane”. Articles published between June 1, 2010, and June 1, 2020, were considered for inclusion. Several post-hoc analyses of randomised clinical trials that investigated the use of peripheral blood biomarkers to predict response to rituximab were identified, but none of them proved effective for patient stratification in clinical practice. Only a few observational studies provided a direct analysis of the disease tissue (ie, the synovial membrane) before treatment with rituximab. Although specific cellular subpopulations and molecular signatures were found to be associated with response, no firm conclusions could be made in relation to the prediction of treatment response, mainly because of the small sample sizes and absence of randomisation.

**Added value of this study**

R4RA is the first biopsy-driven, multicentre, randomised trial comparing tocilizumab with rituximab in patients with rheumatoid arthritis who had inadequate responses to anti-TNF drugs stratified for synovial B-cell status. Although there was no significant difference between the rituximab and the tocilizumab groups using histological B-cell classification for the primary endpoint, the study showed a significant difference in favour of tocilizumab in the number of patients with an improvement in Clinical Disease Activity Index (CDAI) by 50% or more and CDAI less than 10·1 (ie, low disease activity, defined as major treatment response; CDAI-MTR). Moreover, when classification was done with RNA sequencing, tocilizumab was superior to rituximab both for the primary outcome and CDAI-MTR. In patients who were classified as B-cell poor, tocilizumab was also superior to rituximab for most secondary outcomes.

**Implications of all the available evidence**

The R4RA trial represents a milestone in the mechanistic investigation at the disease tissue level of the relationship between drug mode of action and clinical response. Compared with the current clinical approach, R4RA shows that in patients with low or absent of B cell expression signature in synovial tissue an alternative treatment—such as IL-6 receptor inhibition with tocilizumab—is superior to B-cell targeting with rituximab. Replication of the results and validation of RNA sequencing classification in independent cohorts might lead to the integration of molecular pathology into clinical algorithms to guide treatment allocation of target biological therapies according to drug target expression levels in the disease tissue.

## Introduction

Rheumatoid arthritis is a chronic immune mediated inflammatory disease characterised by synovitis and joint damage that results in considerable morbidity and increased mortality.^1^ Biological disease modifying antirheumatic drugs (DMARDs) have transformed the outlook for patients with rheumatoid arthritis; however, the absence of a meaningful response to treatment in approximately 40% of patients and the potential side-effects and high cost of biological DMARDs have underlined the need to identify predictive markers of response to facilitate patient stratification before treatment and ensure the best therapeutic outcome.^2^

B cells are known to contribute to the pathogenesis of rheumatoid arthritis by driving synovial inflammation through the production of local disease specific autoantibodies,^3^ secreting proinflammatory and osteoclastogenic cytokines,^4^ and acting as antigen presenting cells. The important role of B cells is also supported by the efficacy of the specific CD20 B-cell depleting drug, rituximab.^5^ Rituximab is licensed for use in rheumatoid arthritis after unsuccessful conventional synthetic DMARDs and tumour necrosis factor (TNF) inhibitor therapy.

However, in this more therapy-resistant patient cohort, clinical response to rituximab is heterogeneous, with only 30% of patients reported to have a 50% improvement in the American College of Rheumatology response criteria (ACR50) at 6 months.^6^ Given the mechanism of action of rituximab, it was hypothesised that the number of circulating B cells before and after treatment could be used to predict treatment response. However, pretreatment peripheral B-cell numbers or depletion levels, measured by conventional flow cytometry, found no association with clinical outcome.^5^, ^6^, ^7^ Moreover, although the depth of depletion measured by high-sensitive flow cytometry initially appeared to be more informative, small studies have reported contradictory results.^8^, ^9^ Nevertheless, these studies have highlighted that a high number of circulating CD20 negative plasmablasts and preplasma cells was associated with non-response. However, the crucial question remains unanswered: why—despite the profound depletion of peripheral blood B cells induced by rituximab in most patients—do only approximately half of patients respond to this therapy? How the number and depletion of B cells in the disease tissue (synovium) relates to rituximab response is also unclear. In that context, variable results from small, observational, biopsy-based studies have been reported.^10^, ^11^, ^12^ In particular, the number of synovial CD79a B cells reported before treatment,^12^ and the reduction of specific molecular signatures^11^ and synovial plasma cells after treatment^10^ have shown an association with response to rituximab. However, the observational nature of these studies, the small number of patients analysed, and the use of different timepoints for assessment of treatment outcome has made drawing firm conclusions difficult.

Of note, as previously shown in joint replacement tissue from late stage rheumatoid arthritis disease,^13^ and in active early rheumatoid arthritis^14^ more than 50% of patients show low or absent B-cell infiltration (B-cell poor) in the synovial biopsy, suggesting that in B-cell poor patients joint inflammation is driven by other cell types. This prompted us to use a randomised trial to test the hypothesis that in patients with low or absent of CD20 B cells in synovial biopsy rituximab would be less efficacious than an alternative targeting biological DMARD (eg, tocilizumab, a specific IL6-receptor inhibitor).

The aim of this study—the first biopsy-driven randomised clinical trial in rheumatoid arthritis—was to evaluate whether tocilizumab is superior to rituximab in improving clinical outcomes in patients with low absent of synovial B cells. The trial represents a milestone, towards precision rheumatology,^15^ through its mechanistic investigation at the disease tissue level of the relationship between drug mode of action and clinical response.

## Methods

### Study design and participants

We did a phase 4, open-label, multicentre, randomised trial in 19 centres from five European countries: the UK, Belgium, Italy, Portugal, and Spain. The study was done in compliance with the Declaration of Helsinki, International Conference on Harmonisation Guidelines for Good Clinical Practice, and local country regulations. The final protocol, amendments, and documentation of consent were approved by the institutional review board of each study centre or relevant independent ethics committees. The study protocol has been published online.

Patients aged 18 years or older who fulfilled the 2010 ACR and European League Against Rheumatism (EULAR) classification criteria for rheumatoid arthritis^16^ and were eligible for treatment with rituximab therapy according to UK National Institute for Health and Care Excellence (NICE) guidelines (patients who had received previous unsuccessful treatment with or were intolerant to conventional synthetic DMARD therapy and at least one biological therapy, excluding the study drugs, rituximab and tocilizumab)^17^ were eligible for inclusion in the study. Patients were identified through rheumatology outpatient clinics at each study site. A full list of the inclusion and exclusion criteria is provided in the appendix (p 1). All patients provided written informed consent.

### Randomisation and masking

At week 0, patients were randomly assigned (1:1) in block sizes of six and four to the rituximab group or the tocilizumab group stratified into four blocks according to histological classification of baseline synovial biopsy (B-cell poor, B-cell rich, germinal centre positive, or unknown) and by site (Queen Mary University London, London, UK *vs* all other sites) using an interactive web response system. The randomisation list and allocation algorithm were prepared by the trial statistician and securely embedded with the application code so that it was not accessible to end users. The programmer was responsible for implementing the allocation algorithm into the randomisation database. The trial manager and trial management team were responsible for checking patient eligibility and doing the randomisation procedure centrally. The randomisation result was sent electronically to all the clinical trial site staff by the trial office except the named joint assessor (research nurse or assistant) at each site, who remained masked to study drug allocation. All site teams remained masked to histological subtypes throughout the duration of the study.

### Procedures

Patients underwent a synovial biopsy of a clinically active joint at entry to the trial. The biopsy was done according to local expertise, with either a US-guided or arthroscopic procedure as previously described.^18^, ^19^ Six to eight biopsies were immediately fixed in 4% paraformaldehyde for paraffin embedding and an additional six immersed in RNA-Later (Ambion) for later RNA extraction and shipped to the Pathology Laboratory, Barts Health NHS Trust, London, UK, for processing and central evaluation, as per protocol standard operating procedure.

A minimum of six synovial biopsies were embedded in paraffin en masse and assessed histologically. The sections were then stained for haematoxylin and eosin and immune-histochemical markers (appendix p 13), as previously described.^3^, ^20^ Sections underwent semi-quantitative scoring to determine expression of CD20 B cells, CD3 T cells, CD138 plasma cells, and CD68 lining and sublining macrophages (appendix p 13). The scoring process was adapted from a previously described and validated score.^20^, ^21^, ^22^ Following histological assessment of CD20 semi-quantitative scores, patients were classified as B-cell rich or B-cell poor at Barts Health NHS Trust by a consultant pathologist (HR), which was confirmed in an independent evaluation by a second expert in synovial pathology, according to a validated algorithm (appendix p 14). Synovial tissues with a CD20 score less than two were classified as B-cell poor; tissues with CD20 score of two to four and CD20 B-cell aggregates were classified as B-cell rich. Any discrepancies in classification were resolved through mutual agreement (between HR and GT). Patients were classified as unknown if definite synovial tissue could not be identified. B-cell rich samples were classified as germinal centre positive if CD21 follicular dendritic cell networks were present (appendix p 14). As predefined in the study protocol, only patients classified as B-cell rich or B-cell poor were included in the primary analysis.

A minimum of six synovial samples per patient were immediately immersed in RNA-Later for RNA sequencing. The RNA from these samples was extracted as described in the appendix (p 15)^20^ and sequenced at Genewiz (Bishop's Stortford, UK) according to the standard operating procedure (appendix p 15). 184 paired-end RNA sequenced samples of 150 base pairs were trimmed to remove the Illumina adaptors with bbduk from the BBMap package (version 37.93) according to default parameters, using R (version 3.6.0). Transcripts were quantified using Salmon (version 0.13.1)^23^ and an index generated from the Gencode (release-29) transcriptome, in accordance with the standard operating procedure. Tximport (version 1.13.10) was used to aggregate the transcript level expression data, counts were then subject to variance stabilising transformation using DESEQ2 (version 1.25.9).^24^ Patients were classified as B-cell poor or B-cell rich according to a previously developed B-cell specific gene module, derived from analysis of FANTOM5 gene expression data.^25^ Because no predetermined cutoff points for B-cell transcript classification were found in the literature, patients were classified as B-cell poor or B-cell rich according to the median transcript module value to avoid potential bias (appendix p 15).

Following synovial biopsy and subsequent random assignment, patients in the rituximab group received two 1000 mg rituximab infusions 2 weeks apart. Patients in the tocilizumab group received 8 mg/kg tocilizumab monthly infusions. Both drugs were obtained from hospital stocks. Patients were followed up every 4 weeks throughout the 48-week trial treatment period during which clinical and safety data were collected (appendix p 17). Clinical outcomes up to week 16 are presented herein.

### Outcomes

The primary endpoint was the difference in Clinical Disease Activity Index (CDAI)^26^ by 50% or more improvement (CDAI50%) rate at 16 weeks between the tocilizumab group and rituximab group in the intention-to-treat population. Primary efficacy analysis evaluated the number of patients meeting the primary endpoint. Patients could be deemed non-responders (predefined in the protocol) if they had CDAI50% but did not have low disease activity, with a CDAI of less than 10·1 (CDAI-major treatment response; CDAI-MTR); thus, a supplementary efficacy analysis in line with the International Council for Harmonisation Guidelines (2019) was done to evaluate the number of patients meeting CDAI-MTR. In addition, as predefined in the protocol, an aim of the trial was also to test both cellular and molecular signatures in the synovial tissue; therefore, CDAI50% and CDAI-MTR were also evaluated in patients classified according to the RNA sequencing.

The study was not powered to evaluate comparative efficacy of either drug in the B-cell rich cohort; however, assessment of CDAI50% response and CDAI-MTR at 16 weeks was done as a supplementary analysis where the response rate of rituximab was compared with tocilizumab. Additional secondary efficacy analyses included assessment of CDAI remission; Disease Activity Score (DAS28)-erythrocyte sedimentation rate (ESR) and DAS28-C reactive protein (CRP) moderate or good EULAR response; DAS28-ESR and DAS28-CRP low-disease activity; DAS28-ESR and DAS28-CRP remission; and patient reported outcomes, such as fatigue (appendix p 2). In addition, to assess the performance of the RNA sequencing stratification method, as a post-hoc analysis we evaluated the RNA sequencing FANTOM5 B cell module by varying the cutoff between B-cell rich and B-cell poor tissue to determine whether the median value was optimal (appendix p 16). The CDAI50% rates of response in patients classified as anti-citrullinated protein antibodies (ACPA) and rheumatoid factor (RF) positive and negative were compared in a post-hoc analysis.

The incidence and severity of treatment and procedure emergent adverse events were monitored throughout the study; adverse event coding was done according to the Medical Dictionary for Regulatory Activities (version 22). The causality and expectedness of all serious adverse events in relation to the trial treatment was assessed by the principal investigator (or delegated medic), according to the severe adverse event definition. If a severe adverse event related to the treatment was unexpected it was considered a suspected unexpected serious adverse reaction. All severe adverse events up to week 48 (and up to 30 days later) were reported by relatedness using the Medical Dictionary for Regulatory Activities lowest level term classification. All adverse events up to week 48 (and up to 30 days later) were reported using the Medical Dictionary for Regulatory Activities system organ class classification. Recurrent events (events that occurred more than once in the same participant) were considered as one event.

### Statistical analysis

A sample size of 82 patients who were B-cell poor was needed to provide 90% power to detect a 35% difference (assuming 55% response rate to tocilizumab and 20% to rituximab (unpublished data) in patients classified as responders by the primary endpoint. The assumed proportion of patients recruited who were B-cell poor was 60%, B-cell rich was 35%, and germinal centre positive was 5%. After estimating that 10% of biopsy samples would be ungradable and assuming a 5% dropout rate, a total of 160 patients would be required to recruit 82 patients who were B-cell poor. No power calculation was done for patients who were B-cell rich.

The primary endpoint and other binary endpoints were analysed using a χ^2^ or Fisher's exact test. For continuous outcomes, an analysis of covariance (ANCOVA) was done, with treatment as factor and baseline value as the continuous covariate. When the assumptions for the ANCOVA were not met, non-parametric ANCOVA was used. Changes from baseline within groups were analysed with a paired Wilcoxon test.

Although the study was not powered to evaluate comparative efficacy of either drug in patients who were B-cell rich, we tested whether rituximab was as efficacious as tocilizumab as a secondary analysis. The analysis of the interaction between treatments and pathotypes was done through the likelihood ratio test between two nested logistic regression models: one with pathotype and treatment as covariates and the other with pathotype, treatment, and their interaction as covariates.

All efficacy analyses were done in the intention-to-treat population and then on the per-protocol set to assess the robustness of the results. The per-protocol population included all patients from the intention-to-treat population who did not have any major protocol violations (appendix p 12). The list of deviations that excluded a patient from the per-protocol population was reviewed at a classification meeting before the data lock. Safety analyses were done on the safety population (intention-to-treat population, including participants who received at least one dose of the trial medication). In the safety analysis, patients were analysed according to their actual treatment in case this differed from the scheduled treatment (randomised or switched). Missing values assumed to be missing at random were imputed using Multiple Imputation by Chained Equations and implemented using Amelia (version 1.7.5; R package).

All statistical analyses were done using R (version 3.5.1). The trial is registered on the ISRCTN database, ISRCTN97443826, and EudraCT, 2012-002535-28. An independent Data Monitoring Ethics Committee met every 6 months during the trial to review the accruing trial data, assess whether there were any safety issues, and to make recommendations to the trial steering committee.

### Role of the funding source

Funding for this study was provided by the Efficacy and Mechanism Evaluation programme of the National Institute for Health Research (NIHR). The funder of the study had no role in study design, data collection, data analysis, data interpretation, or writing of the Article. No industry funding was implicated in this study.

## Results

Between Feb 28, 2013, and Jan 17, 2019, 212 patients were screened, of whom 190 (89%) gave consent, 167 (79%) had synovial biopsies, and 164 (77% [131 women]) were randomly assigned to one of the two treatment groups. Three patients were randomly assigned, but did not receive study drug (individuals were not included in the intention-to-treat analysis). The trial ended as recruitment targets were reached. After synovial biopsies were classified, 83 (50%) patients were randomly assigned to the rituximab group and 81 (50%) to the tocilizumab group. 81 (99%) of the 82 patients from the rituximab group and 73 (92%) of the 79 patients in the tocilizumab group completed treatment to primary endpoint at week 16 (figure). 62 (38%) of the 164 included patients were recruited at Barts Health NHS Trust (appendix p 3).

**Figure:**
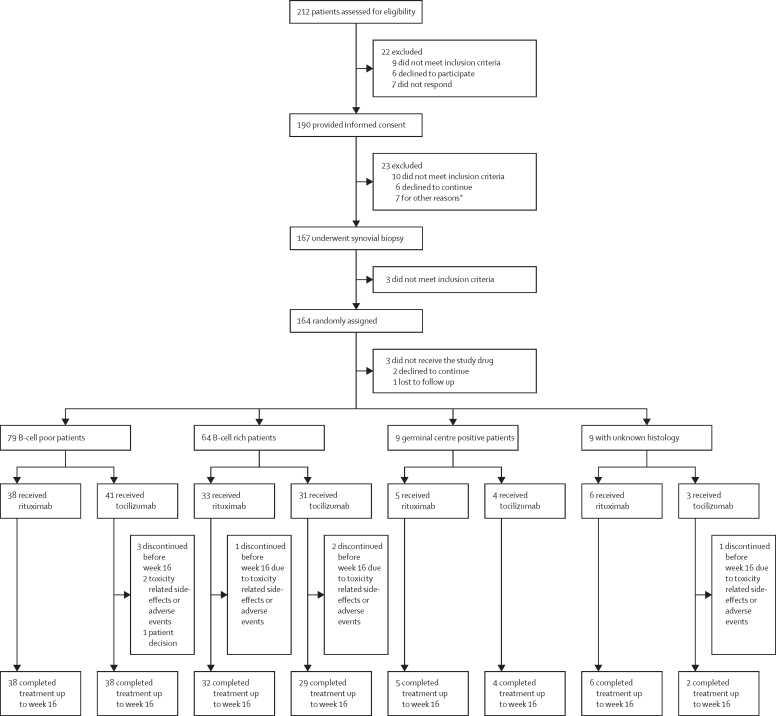
Trial profile *Six patients did not have suitable joints at biopsy and one for clinical reasons unrelated to rheumatoid arthritis.

Baseline characteristics, disease activity, and histological groups are reported in table 1 and the appendix (pp 4–5). 79 (49%) of 161 patients were histologically classified as B-cell poor, 64 (40%) patients were B-cell rich, 9 (6%) were germinal centre positive, and 9 (6%) were unknown. Of the 79 (49%) of 161 patients classified as B-cell poor, 38 (48%) were assigned to the rituximab group and 41 (52%) to the tocilizumab group.

**Table 1:** Baseline characteristics of patients, stratified by histological classification and investigational medicinal product

	**Overall**				**B-cell poor**				**B-cell rich**				
		All patients (n=161)	Rituximab (n=82)	Tocilizumab (n=79)	p value	All patients (n=79)	Rituximab (n=38)	Tocilizumab (n=41)	p value	All patients (n=64)	Rituximab (n=33)	Tocilizumab (n=31)	p value
Sex													
	Female	128 (80%)	62 (76%)	66 (84%)	0·21	69 (87%)	32 (84%)	37 (90%)	0·42	50 (78%)	23 (70%)	27 (87%)	0·092
	Male	33 (20%)	20 (24%)	13 (16%)	0·21	10 (13%)	6 (16%)	4 (10%)	0·42	14 (22%)	10 (30%)	4 (13%)	0·092
Age, years		55·5 (47·4–65·3)	55·7 (47·7–65·5)	55·5 (47·3–65·1)	0·81	56·3 (47·2–65·9)	57·9 (47·0–68·9)	54·7 (47·3–64·3)	0·54	55·3 (48·1–64·6)	54·1 (47·1–63·1)	57·4 (51·3–67·4)	0·22
Median disease duration, years		9·0 (4·0–19·0)	9·50 (4·0–20·7)	9·00 (4·0–18·0)	0·71	11·0 (6·0–22·5)	11·0 (6·0–24·7)	12·0 (5·0–21·0)	0·72	7·0 (4·0–15·2)	6·0 (4·0–16·0)	7·0 (4·5–13·5)	0·76
Clinical disease activity index		29·8 (21·7–40·6)	30·6 (22·8–40·6)	29·4 (21·5–40·3)	0·67	30·4 (22·3–39·8)	30·8 (23·5–38·4)	30·4 (22·3–44·2)	0·64	29·0 (21·1–40·9)	28·9 (20·7–40·7)	31·4 (21·6–41·1)	0·72
ESR (mm/h)		31·0 (17·0–48·0)	34·5 (17·0–48·0)	28·0 (18·5–46·5)	0·57	29·0 (17·0–44·0)	30·5 (16·0–44·5)	28·0 (19·0–42·0)	0·91	34·5 (19·0–54·0)	41·0 (19·0–54·0)	32·0 (19·0–55·5)	0·71
CRP (mg/L)		11·0 (5·0–27·0)	10·0 (5·0–23·0)	15·0 (5·0–32·0)	0·29	9·0 (2·5–18·0)	7·5 (1·0–15·7)	13·0 (6·0–23·0)	0·098	16·5 (7·7–33·7)	14·0 (8·0–32·0)	18·0 (6·0–44·0)	0·83
RF or ACPA positive		140 (87%)	73 (89%)	67 (85%)	0·42	67 (85%)	34 (89%)	33 (80%)	0·26	55 (86%)	28 (85%)	27 (87%)	0·79
	RF positive	119 (74%)	64 (78%)	55 (70%)	0·22	55 (70%)	29 (76%)	26 (63%)	0·21	49 (77%)	26 (79%)	23 (74%)	0·66
	ACPA positive	128 (80%)	67 (82%)	61 (77%)	0·48	61 (77%)	30 (79%)	31 (76%)	0·72	50 (78%)	26 (79%)	24 (77%)	0·89
Haemoglobin (g/L)		123·0 (110·5–131·5)	121·0 (109·0–131·0)	123·0 (111·5–131·7)	0·58	123·0 (110·5–129·0)	123·0 (109·5–129·0)	123·0 (111·0–129·0)	0·99	120·0 (109·2–131·5)	120·0 (108·5–132·0)	119·5 (110·2–130·0)	0·855
Number of tender joints (0–28)		11·0 (6·0–18·0)	10·5 (6·2–18·7)	11·0 (6·0–16·0)	0·63	11·0 (7·0–18·0)	11·0 (7·0–17·7)	12·0 (7·0–18·0)	0·85	10·0 (6·0–17·2)	10·0 (6·0–19·0)	12·0 (6·0–15·5)	0·824
Number of swollen joints (0-28)		6·0 (3·0–10·0)	6·00 (4·0–9·0)	6·0 (3·0–10·5)	0·76	6·0 (4·0–10·5)	5·0 (3·2–8·7)	7·0 (4·0–12·0)	0·17	6·0 (3·7–9·3)	6·0 (4·0–9·0)	6·0 (3·5–10·0)	0·984
DAS28-ESR		5·81 (1·25)	5·84 (1·19)	5·78 (1·31)	0·74	5·88 (1·10)	5·80 (1·04)	5·95 (1·17)	0·56	5·85 (1·32)	5·76 (1·41)	5·94 (1·23)	0·581
DAS28-CRP		5·31 (1·20)	5·30 (1·15)	5·33 (1·26)	0·90	5·28 (1·13)	5·13 (1·02)	5·41 (1·23)	0·27	5·40 (1·27)	5·33 (1·30)	5·48 (1·24)	0·639
Ultrasound 12-max score (Power Doppler)		4·0 (1·0–9·0)	4·0 (0·2–8·0)	6·0 (1·5–10·0)	0·44	4·0 (0·0–8·0)	4·0 (3·0–7·0)	2·0 (0·0–8·0)	0·39	6·0 (2·0–10·5)	6·0 (1·5–9·0)	7·0 (4·0–12·2)	0·289
Ultrasound 12-max score (synovial thickening)		15·0 (11·5–22·0)	16·0 (13·0–22·0)	15·0 (10·0–20·2)	0·41	14·0 (9·0–18·0)	14·0 (9·5–17·5)	13·5 (7·2–19·2)	0·59	19·0 (14·5–24·5)	19·0 (15·0–22·0)	19·0 (14·0–25·0)	0·934
SHSS, total		8·5 (2·5–39·6)	8·5 (2·5–39·6)	9·3 (2·5–36·9)	0·89	6·0 (1·0–39·6)	6·0 (1·0–37·0)	6·0 (1·0–50·0)	0·64	10·8 (3·5–51·0)	12·5 (2·9–64·5)	10·5 (5·3–24·3)	0·666
	SHSS, joint space narrowing	6·8 (1·1–29·6)	6·3 (1·3–29·6)	7·0 (1·4–29·0)	0·82	3·0 (0·3–28·5)	2·5 (0·0–23·0)	5·00 (1·0–30·5)	0·41	8·8 (2·3–34·5)	8·0 (2·8–43·1)	9·8 (2·9–20·1)	0·756
	SHSS, erosion	1·5 (0·0–8·9)	3·3 (0·1–8·9)	0·8 (0·0–8·4)	0·35	1·8 (0·0–10·5)	3·5 (0·0–9·0)	1·5 (0·0–11·0)	0·80	1·5 (0·0–6·9)	2·5 (0·4–16·8)	0·8 (0·0–5·4)	0·401
Previous methotrexate use		161 (100%)	82 (100%)	79 (100%)	NA	79 (100%)	38 (100%)	41 (100%)	NA	64 (100%)	33 (100%)	31 (100%)	NA
Previous prednisolone use		90 (56%)	44 (54%)	46 (58%)	0·56	42 (53%)	23 (61%)	19 (46%)	0·21	40 (62%)	18 (55%)	22 (71%)	0·175
Number of previous biological drugs used, (%; anti-TNF *vs* other*)†		..	..	..	0·19	..	..	..	0·15	..	..	..	0·711
	One	116 (72%; 116 *vs* 0)	62 (76%; 62 *vs* 0)	54 (68%; 54 *vs* 0)	NA	52 (66%; 52 *vs* 0)	25 (66%; 25 *vs* 0)	27 (66%; 27 *vs* 0)	NA	50 (78%; 50 *vs* 0)	27 (82%; 27 *vs* 0)	23 (74%; 23 *vs* 0)	NA
	Two	36 (22%; 32 *vs* 4]	14 (17%; 11 *vs* 3)	22 (28%; 21 *vs* 1)	NA	21 (27%; 19 *vs* 2)	8 (21%;6 *vs* 2)	13 (32%; 13 *vs* 0)	NA	11 (17%; 9 *vs* 2)	5 (15%;4 *vs* 1)	6 (19%; 5 *vs* 1)	NA
	Three or more	9 (6%;5 *vs* 4)	6 (7%;3 *vs* 3)	3 (4%;2 *vs* 1)	NA	6 (8%;3 *vs* 3)	5 (13%;2 *vs* 3)	1 (2%;1 *vs* 0)	NA	3 (5%;2 *vs* 1)	1 (3%;1 *vs* 0)	2 (6%;1 *vs* 1)	NA

Data are n (%), median [IQR], or mean (SD). ACPA=anti-citrullinated protein antibody. CRP=C-reactive protein. DAS28=28 joint count Disease Activity Score. ESR=erythrocyte sedimentation rate. RF=rheumatoid factor. NA=not applicable. SHSS=Sharp van der Heijde score. TNF=tumour necrosis factor.

* Eight patients in total used non-TNF inhibitor biological drugs (seven received abatacept and one received vaccine RA TNF-K-006 for a clinical study).

† p values refer to the χ^2^ comparing the number of biological drugs used in rituximab and tocilizumab groups.

At 16 weeks in the B-cell poor population, there was no statistically significant difference in rate of CDAI50% response between the rituximab group (17 [45%] of 38 patients) and the tocilizumab group (23 [56%] of 41 patients; difference 11% [95% CI −11 to 33], p=0·31; table 2). However, a predefined supplementary analysis of CDAI-MTR did show a statistical significance between the rituximab group (9 [24%] of 38 patients) and the tocilizumab group (19 [46%] of 41 patients; difference 22% [2 to 43], p=0·035; table 2). In addition, the response rates in the B-cell poor patients in the tocilizumab group were statistically significantly higher for a number of secondary endpoints (table 2).

**Table 2:** Clinical outcomes at 16 weeks in the intention-to-treat B-cell poor population

	**Histological classification**				**RNA sequencing classification**			
	Rituximab (n=38)	Tocilizumab (n=41)	Treatmenteffect	Unadjusted p value	Rituximab (n=33)	Tocilizumab (n=32)	Treatmenteffect	Unadjusted p value
Primary endpoint*								
CDAI ≥50% improvement at week 16	17 (45%)	23 (56%)	11% (−11 to 33)	0·31	12 (36%)	20 (63%)	26% (3 to 50)	0·035
Supplementary endpoint*								
CDAI ≥50% improvement and CDAI ≤10·1 at week 16	9 (24%)	19 (46%)	23% (2 to 43)	0·035	4 (12%)	16 (50%)	38% (17 to 59)	0·0012
Binary secondary endpoints*								
CDAI ≤10·1 at week 16	11 (29%)	19 (46%)	17% (−3 to 38)	0·11	5 (15%)	16 (50%)	35% (14 to 56)	0·0036
DAS28-ESR ≤3·2 at week 16	10 (26%)	18 (44%)	18% (−4 to 38)	0·10	6 (18%)	17 (53%)	35% (13 to 57)	0·0032
DAS28-CRP ≤3·2 at week 16	12 (32%)	19 (46%)	15% (−7 to 36)	0·18	7 (21%)	16 (50%)	29% (7 to 51)	0·015
DAS28-ESR ≤2·6 at week 16	6 (16%)	15 (37%)	21% (2 to 40)	0·037	3 (9%)	13 (41%)	32% (12 to 51)	0·004
DAS28-CRP ≤2·6 at week 16	7 (18%)	13 (32%)	13% (−6 to 32)	0·17	4 (12%)	10 (31%)	19% (0 to 39)	0·076
Moderate or good EULAR DAS28-ESR response at week 16	25 (66%)	36 (88%)	22% (4 to 40)	0·031	21 (64%)	30 (94%)	30% (12 to 49)	0·0053
Moderate or good EULAR DAS28-CRP response at week 16	22 (58%)	32 (78%)	20% (0 to 40)	0·054	18 (55%)	27 (84%)	30% (9 to 51)	0·015
**Continuous secondary endpoints**†								
CDAI, least squares mean change at week 16	−12·1 (1·9)	−15·7 (1·9)	3·6 (−1·7 to 8·9)	0·18	−10·9 (2·0)	−17·2 (2·0)	6·3 (0·7 to 12·0)	0·029
DAS28-ESR, least squares mean change at week 16	−1·5 (0·2)	−2·6 (0·2)	1·1 (0·5 to 1·7)	0·0006	−1·3 (0·2)	−2·8 (0·2)	1·5 (0·9 to 2·2)	<0·0001
DAS28-CRP, least squares mean change at week 16	−1·3 (0·2)	−2·0 (0·2)	0·7 (0·1 to 1·3)	0·032	−1·1 (0·2)	−2·1 (0·2)	1·0 (0·4 to 1·6)	0·0021
HAQ, least squares mean change at week 16	−0·3 (0·1)	−0·4 (0·1)	0·1 (−0·1 to 0·3)	0·40	−0·2 (0·1)	−0·2 (0·1)	<0·1 (−0·2 to 0·2)	0·91
FACIT, least squares mean change at week 16	1·6 (1·1)	5·6 (1·1)	−4·0 (−7·2 to −0·8)	0·015	2·1 (1·4)	4·9 (1·4)	−2·8 (−6·9 to 1·2)	0·16
SF36-PCS, least squares mean change at week 16	4·1 (1·5)	7·3 (1·5)	−3·2 (−7·4 to 0·9)	0·12	3·5 (1·5)	4·3 (1·5)	−0·8 (−5·0 to 3·5)	0·72
SF36-MCS, least squares mean change at week 16	−0·7 (1·6)	2·1 (1·6)	−2·8 (−7·3 to 1·8)	0·22	0·9 (1·8)	4·5 (1·9)	−3·7 (−8·8 to 1·5)	0·16

Data are n (%) or least squares mean (SD), unless otherwise specified. Patients classified histologically are compared with those categorised with RNA sequencing. CDAI=Clinical Disease Activity Index. CRP=C-reactive protein. DAS28=28 joint count Disease Activity Score. EULAR=European League against Rheumatism. ESR=erythrocyte sedimentation rate. FACIT=Functional Assessment of Chronic Illness Therapy. HAQ=Health Assessment Questionnaire. SF36-MCS=Mental Components Summary of the SF-36 questionnaire. SF36-PCS=Physical Components Summary of the SF-36 questionnaire. SF36=36-Item Short Form Health Survey.

* Treatment effect measured with percentage difference (95% CI).

† Treatment effect measured with least squares mean (95% CI).

Quality of life outcome measures (Functional Assessment of Chronic Illness Therapy/36-Item Short Form Health Survey scores) improved more between baseline and 16 weeks in the tocilizumab group (table 2). We observed little difference in Health Assessment Questionnaire scores between the rituximab group and the tocilizumab group (table 2). Of note, per-protocol analyses were consistent with intention-to-treat outcomes (appendix p 6).

Of the 162 patients with RNA available for extraction, nine (6%) patients were excluded after histological classification as germinal centre positive, one (1%) patient withdrew before drug administration, and 28 (17%) patients were excluded because of RNA sequencing quality control or poor mapping. 124 (77%) patients had RNA sequencing data available for analysis (appendix p 15); 65 (52%) of whom were classified as B-cell poor. The tocilizumab group had a significantly higher response rate compared with the rituximab group for both CDAI50% (rituximab group 12 [36%] of 33 patients *vs* tocilizumab group 20 [63%] of 32 patients; difference 26% [95% CI 2–50], p=0·035) and for CDAI-MTR (rituximab group 4 [12%] of 33 patients *vs* tocilizumab group 16 [50%] of 32 patients; difference 38% [17–59], p=0·0012). The response rates for a number of secondary outcomes were also significantly higher in the tocilizumab group compared with the rituximab group (table 2). Per-protocol analyses were consistent with the intention-to-treat results (appendix p 7).

64 (40%) of the 161 patients categorised histologically and 59 (47%) of the 124 patients categorised with RNA sequencing were classified as B-cell rich. Although the study was not powered for the comparative analysis of B-cell rich populations in each treatment group, we observed similar week 16 response rates between the rituximab group and the tocilizumab group for most of the endpoints analysed, including CDAI50% and CDAI-MTR (table 3). Similar effects were seen for a number of additional secondary endpoints (table 3). Of note, compared with the analysis in the B-cell poor population, there were minimal differences in quality of life measures between the rituximab and tocilizumab groups (table 3). Per-protocol analyses were consistent with the intention-to-treat results (appendix pp 8–9).

**Table 3:** Clinical outcomes at 16 weeks in the intention-to-treat B-cell rich population

	**Histological classification**				**RNA sequencing classification**			
	Rituximab (n=33)	Tocilizumab (n=31)	Treatment effect	Unadjusted p value	Rituximab (n=30)	Tocilizumab (n=29)	Treatment effect	Unadjusted p value
Primary endpoint*								
CDAI ≥50% improvement at week 16	13 (39%)	16 (52%)	12% (−12 to 37)	0·33	15 (50%)	14 (48%)	−2% (−27 to 24)	0·89
Supplementary endpoint*								
CDAI ≥50% improvement and CDAI ≤10·1 at week 16	5 (15%)	11 (36%)	20% (−1 to 41)	0·085	7 (23%)	9 (31%)	8% (−15 to 30)	0·51
Binary secondary endpoints*								
CDAI ≤10·1 at week 16	7 (21%)	12 (39%)	18% (−5 to 40)	0·13	10 (33%)	10 (35%)	1% (−23 to 25)	0·93
DAS28-ESR ≤3·2 at week 16	8 (24%)	13 (42%)	18% (−5 to 40)	0·13	9 (30%)	10 (35%)	5% (−19 to 28)	0·71
DAS28-CRP ≤3·2 at week 16	12 (36%)	13 (42%)	6% (−18 to 30)	0·65	14 (47%)	11 (38%)	−9% (−34 to 16)	0·50
DAS28-ESR ≤2·6 at week 16	2 (6%)	11 (36%)	29% (11 to 48)	0·0047	3 (10%)	10 (35%)	25% (4 to 45)	0·03
DAS28-CRP ≤2·6 at week 16	4 (12%)	9 (29%)	17% (−3 to 36)	0·12	4 (13%)	8 (28%)	14% (−6 to 35)	0·21
Moderate or good EULAR DAS28-ESR response at week 16	25 (76%)	27 (87%)	11% (−8 to 30)	0·34	24 (80%)	24 (83%)	3% (−17 to 23)	1·00
Moderate or good EULAR DAS28-CRP response at week 16	23 (70%)	25 (81%)	11% (−10 to 32)	0·31	23 (77%)	23 (79%)	3% (−19 to 24)	0·81
**Continuous secondary endpoints**†								
CDAI, least squares mean change at week 16	−13·2 (2·1)	−14·2 (2·1)	1·0 (−4·9 to 6·8)	0·73	−15 (2·1)	−14·1 (2·2)	−0·5 (−6·5 to 5·6)	0·88
DAS28-ESR, least squares mean change at week 16	−1·5 (0·2)	−2·6 (0·2)	1·1 (0·5 to 1·8)	0·0009	−1·7 (0·2)	−2·4 (0·2)	0·7 (0·1 to 1·4)	0·026
DAS28-CRP, least squares mean change at week 16	−1·5 (0·2)	−2·0 (0·2)	0·6 (−0·0 to 1·1)	0·059	−1·7 (0·2)	−1·9 (0·2)	0·3 (−0·3 to 0·9)	0·34
HAQ, least squares mean change at week 16	−0·3 (0·1)	−0·4 (0·1)	0·1 (−0·2 to 0·4)	0·41	−0·3 (0·1)	−0·5 (0·1)	0·2 (−0·0 to 0·5)	0·085
FACIT, least squares mean change at week 16	8·5 (1·9)	7·8 (2·0)	0·7 (−4·8 to 6·3)	0·79	6·9 (1·8)	8·34 (1·9)	−1·5 (−6·7 to 3·8)	0·58
SF36-PCS, least squares mean change at week 16	7·0 (2·0)	8·5 (2·1)	−1·5 (−7·2 to 4·2)	0·59	6·9 (1·9)	10·9 (2·1)	−4·0 (−9·6 to 1·6)	0·16
SF36-MCS, least squares mean change at week 16	5·4 (2·2)	3·3 (2·4)	2·1 (−4·5 to 8·5)	0·53	4·4 (2·3)	3·01 (2·5)	1·4 (−5·4 to 8·2)	0·68

Data are n (%) or least squares mean (SD), unless otherwise specified. Patients classified histologically are compared with those categorised with RNA sequencing. CDAI=Clinical Disease Activity Index. CRP=C-reactive protein. DAS28=28 joint count Disease Activity Score. EULAR=European League against Rheumatism. ESR=erythrocyte sedimentation rate. FACIT=Functional Assessment of Chronic Illness Therapy. HAQ=Health Assessment Questionnaire. SF36-MCS=Mental Components Summary of the SF-36 questionnaire. SF36-PCS=Physical Components Summary of the SF-36 questionnaire. SF36=36-Item Short Form Health Survey.

* Treatment effect measured with percentage difference (95% CI).

† Treatment effect measured with least squares mean (95% CI).

Logistic regression analysis showed no evidence of an interaction between either study drug and histologically defined B cell subgroups for primary endpoint, but a statistically significant interaction between RNA sequencing-defined B cell subgroup and the study drugs was reported (p=0·049) when using CDAI-MTR. However, when differences in CDAI50% response rates to rituximab between patients classified histologically as B-cell rich or B-cell poor were evaluated, no statistically significant differences in outcome (p=0·81) were reported.

In the rituximab there was no statistically significant difference in CDAI50% response rates between those classified as ACPA-positive (30 [45%] of 67 patients) and ACPA-negative (7 [46%] of 15 patients, p=0·89] and between patients classified as RF-positive (28 [43%] of 64 patients) and RF-negative (9 [50%] of 18 patients, p=0·63; appendix p 10). There was also no significant difference in response rates according to RF and ACPA seropositive patients treated with tocilizumab (appendix p 10).

Safety data up to 48 weeks are summarised in table 4 and in the appendix (p 11). Occurrence of adverse events (rituximab group 76 [70%] of 108 patients *vs* tocilizumab group 94 [80%] of 117 patients; difference 10% [95% CI −1 to 21]) and serious adverse events (rituximab group 8 [7%] of 108 patients *vs* tocilizumab group 12 [10%] of 117 patients; difference 3% [–5 to 10]) was not significantly different between treatment groups. One death due to suicide was reported in the rituximab group. No malignancies were reported within the 48-week trial period. Two patients in the rituximab group (corneal melt [reported as a suspected unexpected serious adverse reaction] and suicide) and three patients in the tocilizumab group (pleural effusion, chest pain, and cytokine release syndrome) discontinued the study regimens because of serious adverse events. Three patients were randomly assigned, but did not receive study drug; no serious adverse events were reported in these patients. Of note, there were no serious adverse events reported related to synovial biopsy.

**Table 4:** Safety data from baseline to 48 weeks

	**Total (n=225)**	**Rituximab (n=108)**	**Tocilizumab (n=117)**	**Percentage difference (95% CI)**
Serious adverse events	20 (9%)	8 (7%)	12 (10%)	3% (−5 to 10)
Serious adverse events related to study drug	12 (5%)	4 (4%)	8 (7%)	3% (−3 to 9)
Abdominal pain	1 (<1%)	1 (1%)	0	NA
Chest pain*	1 (<1%)	0	1 (1%)	NA
Chronic obstructive pulmonary disease exacerbation	1 (<1%)	1 (1%)	0	NA
Corneal melt*	1 (<1%)	0	1 (1%)	NA
Cytokine release syndrome†	1 (<1%)	1 (1%)	0	NA
Dental cyst	1 (<1%)	0	1 (1%)	NA
Diarrhoea	1 (<1%)	1 (1%)	0	NA
Lower respiratory tract infection	1 (<1%)	0	1 (1%)	NA
Leg pain	1 (<1%)	0	1 (1%)	NA
Pilonidal sinus	1 (<1%)	0	1 (1%)	NA
Pneumonia	1 (<1%)	0	1 (1%)	NA
Urinary tract infection	1 (<1%)	0	1 (1%)	NA
Serious adverse events unrelated to study drug	14 (6%)	4 (4%)	10 (9%)	5% (−1 to 11)
Chest pain	2 (1%)	1 (1%)	1 (1%)	0% (−3 to 2)
Chest pain (cardiac)	1 (<1%)	0	1 (1%)	NA
Coronary angiogram	1 (<1%)	0	1 (1%)	NA
Drainage of pilonidal abscess	1 (<1%)	1 (1%)	0	NA
Hallux valgus	1 (<1%)	0	1 (1%)	NA
Parathyroid adenoma	1 (<1%)	0	1 (1%)	NA
Pleural effusion†	1 (<1%)	0	1 (1%)	NA
Seizure	1 (<1%)	0	1 (1%)	NA
Suicide†	1 (<1%)	1 (1%)	0	NA
Toe amputation	1 (<1%)	0	1 (1%)	NA
Total knee replacement	1 (<1%)	1 (1%)	0	NA
Urinoma	1 (<1%)	0	1 (1%)	NA
Serious adverse events resulting in study drug discontinuation	5 (2%)	2 (3%)	3 (3%)	1% (−3 to 5)
Any non-serious adverse event	170 (76%)	76 (70%)	94 (80%)	10% (−1 to 21)

Data are n (%). All events reported after the first prescription of the study drug up to week 48 (+30 days). Some patients had more than one adverse event. Events are classified using the Medical Dictionary for Regulatory Activities system classification, using the lowest level terms grouping. No cancer was observed during the treatment period. However, there was one kidney carcinoma after week 48. NA=not applicable.

* Event was a suspected unexpected serious adverse reaction.

† Event led to treatment drug discontinuation.

## Discussion

Rituximab remains an important therapeutic option for patients with rheumatoid arthritis; however, clinical response remains heterogeneous, with only 30% of patients with an inadequate response to anti-TNF reported to have an ACR50 response at 6 months.^6^ The mechanism of response and non-response remains unknown. Thus, understanding these mechanisms is crucial to avoid unnecessary exposure to a potentially toxic drug and delay in bringing disease under control. Because more than 50% of patients with rheumatoid arthritis show low or absent B-cell infiltration in the main disease tissue (joint synovium), the rituximab versus tocilizumab in anti-TNF inadequate responder patients with rheumatoid arthritis (R4RA) trial was designed and independently supported by the UK NIHR to determine whether target expression levels (CD20 B cells) and B-cell associated molecular signatures in the synovial tissue can provide a mechanistic explanation for drug mode of action and treatment response.

In this first biopsy-based, multicentre, randomised controlled trial in rheumatoid arthritis, we tested the hypothesis that, in patients stratified for low or absent synovial-biopsy CD20 B cells—the target for rituximab—tocilizumab, a specific IL-6-receptor inhibitor, would be superior. In patients classified as B-cell poor by histological classification, there was no statistically significant difference between the two treatment groups for the primary endpoint: CDAI50%. However, tocilizumab was superior to rituximab in patients with low disease activity, defined as CDAI50% and CDAI-MTR (CDAI <10·1).

In addition, when patients were classified as B-cell poor or B-cell rich by RNA sequencing, both the primary endpoint and CDAI-MTR reached statistical significance in the B-cell poor group. The statistically significant interaction between RNA sequencing and the study drugs observed with CDAI-MTR suggests that the treatment effect difference between rituximab and tocilizumab was statistically different between the RNA sequencing-stratified B-cell poor and B-cell rich groups.

The reasons for the histological and RNA sequencing differences are likely to relate to the sensitivity of the classification technique. CD20 staining was evaluated at three cutting levels on a minimum of six biopsies, which is recommended for use in clinical trials and reported to be representative of the whole joint tissue.^27^ Although the semi-quantitative score used for balanced stratification (before randomisation) had been validated both against digital image analysis and the transcript concentrations, determined using the FANTOM5-derived B-cell related gene set,^20^, ^25^ because no published gold standard is available, the cutoff of 0–1 for B-cell poor classification and 2–4 for B-cell rich classification was set arbitrarily on the basis of a previous pilot study and might not have been at an optimal level for the whole trial. Furthermore, considering that these cutoffs were determined by physically counting the number of CD20 B cells, miscounting had the potential for misclassification.

Classification with RNA sequencing was determined by applying a FANTOM5-derived module, which includes 73 genes associated with B cells.^20^, ^25^ Additionally, the six biopsied were homogenised and pooled to provide a more integrated measure (expression of 30 000 genes) of pathobiological processes within the entire active joint and arguably a more precise estimate of the number of mature CD20 B cells and B cells at different stages of differentiation (eg, plasmablast and preplasma cells). Because plasmablast and preplasma cells, both in the peripheral blood and synovial tissue, have been shown to influence response to rituximab,^9^, ^10^, ^11^, ^12^ the RNA sequencing classification clearly appears to be more sensitive. In addition, the application of RNA sequencing classification overcame a number of limitations associated with histological classification, including the replacement of subjective assessments of synovial B-cell infiltration by histopathology with an objective method using the median transcript expression levels of a B-cell gene set module. Of note, in a post-hoc analysis, the FANTOM5 B cell module median values were confirmed to have performed optimally because varying the cutoffs across a 20% range made no difference to results of the study based on the primary outcome measure.

In patients classified as B-cell poor by RNA sequencing, tocilizumab was significantly superior to rituximab not only in relation to CDAI50% and CDAI-MTR but also in most of the secondary endpoints considered, which suggests a closer association with a broad range of outcome measures. However, in patients classified as B-cell rich by RNA sequencing the efficacy of rituximab overlapped with tocilizumab suggesting that target expression levels in the disease tissue are important mechanistically in determining non-response and response. Namely, in patients classified as B-cell poor, tocilizumab is more efficacious at inhibiting non-B-cell dependent pathways (eg, IL-6), whereas in patients classified as B-cell rich, tocilizumab and rituximab are similarly efficacious at modulating B cell function.

This study also highlighted the potential importance of synovial biopsy in relationship to clinical response and RF and ACPA serological status because no statistically significant difference in clinical response rates to rituximab or tocilizumab were reported between patients who were positive and those who were negative for RF and ACPA. Thus, it is possible that the synovial biopsy might be more sensitive than serology in stratifying patients to rituximab therapy because—although there is a strong association between RF and ACPA positivity and B-cell rich synovitis^14^—there are some patients who are RF and ACPA positive but have low or absent B cells in the joint. However, these results must be interpreted with caution considering the small number of patients who were seronegative in the trial, with a potential for a false-negative finding; most studies have reported better response to rituximab in patients who are RF and ACPA positive.^28^

Regarding safety, although a higher number of serious adverse events and adverse events were reported in the tocilizumab group, these appeared largely unrelated to study drug, and there was no statistically significant difference between the two groups. Of note, there were no serious adverse events related to the synovial biopsy supporting previous data confirming the safety of minimally invasive ultrasound-guided procedure done by rheumatologists.^18^

The study had some inevitable limitations. First, the potentially inaccurate binary B-cell poor or B-cell rich histological classification. Resolution of this issue will require analysis of the trial to determine a precise cutoff or a different classification method (eg, using continuous variable data, such as transcript concentrations) that is a sensitive predictive tool of clinical response marker for other therapeutic targets (eg, PD1).^29^ Second, the choice of tocilizumab as an active comparator to rituximab might not have been optimal because tocilizumab itself modulates B-cell function and survival.^30^ Third, the study design, with no drug allocation double-blinding, might have favoured the faster acting tocilizumab, given as monthly infusion, compared with rituximab, given every 6 months. However, because no study drug funding was provided, tocilizumab was the only choice available according to NICE guidance, and the Ethics Committee advised against double-blinding the trial because it would be impractical and extremely inconvenient for patients. Fourth, despite the washout period for TNF inhibitors and standardisation of steroid and conventional synthetic DMARD therapy at trial entry, it is possible that baseline resistant and responsive pathways were modulated heterogeneously by previous or concomitant therapy. Finally, the choice of an improvement of CDAI50% as a primary binary outcome, rather than EULAR-DAS28-ESR response, illustrates the different sensitivity of the available assessments methods. EULAR-DAS28-ESR response would have led to meeting the primary outcome even by the histological classification.

In conclusion, we report the results from the first pathobiology-driven, stratified, multicentre randomised trial in rheumatoid arthritis, which showed that although the histological classification of rheumatoid arthritis synovial tissue was insensitive in determining treatment response in the primary analysis RNA sequencing stratification had significant associations with clinical responses. Additionally, in patients with low or absent B-cell lineage expression signature (the target for rituximab) tocilizumab was superior to rituximab with regard to the number of patients with a CDAI50% or CDAI-MTR, and for most of the secondary outcomes.

In patients presenting with a B-cell rich synovium, rituximab was as effective as tocilizumab. These results suggest that disease tissue target expression concentrations are important to inform treatment response. However, because of the limitations of the study, the reported findings cannot justify a change in clinical practice. Nonetheless, replication of the results in independent trials (eg, the biopsy-driven Medical Research Council-funded Stratification of Biologic Therapies for Rheumatoid Arthritis by Pathobiology trial; EudraCT 2017-004079-30), and the validation and refinement of the RNA sequencing pathology classification (eg, using continuous variable data rather than a binary classification), might lead to the development of tests of clinical use for treatment allocation of specific targeted biological therapies—according to the corresponding target expression levels in the disease tissue—and towards precision rheumatology.^15^

The ability to target biological therapies to the right patients, rather than continue current practice of trial and error, might improve clinical response and early remission, with a major effect on disability and related health and societal costs while also reducing patient exposure to potentially toxic drugs. This would also align rheumatology practice with other disease in which the integration of molecular pathology into clinical algorithms leads to treatment allocation according to drug target expression levels in the disease tissue as part of routine clinical practice.^31^

## Data sharing

The anonymised raw data will be stored in a non-publicly available repository called TranSMART. All data requests should be submitted to the corresponding author for consideration. Access to anonymised data may be granted following review.
