# Supplementary material for: Rituximab versus tocilizumab in anti-TNF inadequate responder patients with rheumatoid arthritis (R4RA): 16-week outcomes of a stratified, biopsy-driven, multicentre, open-label, phase 4 randomised controlled trial
Source: Lancet. 2021 Jan 23;397(10271):305–17. doi: 10.1016/S0140-6736(20)32341-2 (PMC7829614; doi:10.1016/S0140-6736(20)32341-2)

THE LANCET

Supplementary appendix

This appendix formed part of the original submission and has been peer reviewed. We post it as supplied by the authors.

Supplement to: Humby F, Durez P, Buch MH, et al. Rituximab versus tocilizumab in anti-TNF inadequate responder patients with rheumatoid arthritis (R4RA): 16-week outcomes of a stratified, biopsy-driven, multicentre, open-label, phase 4 randomised controlled trial. *Lancet* 2021; **397:** 305–17.

Appendix R4RA Randomised Clinical Trial: Supplementary Tables and Figures

Table 1: Inclusion/exclusion criteria

Inclusion Criteria
1. Patients who have failed anti-TNF therapy (inadequate responders – ir). Note; this includes patients who have failed anti-TNF therapy because of reactions.<br>2. Who are eligible for Rituximab therapy according to UK NICE guidelines<br>3. Patients should be receiving a stable dose of methotrexate for at least 4 weeks prior to biopsy visit.<br>4. 2010 ACR / EULAR Rheumatoid Arthritis classification criteria for a diagnosis of Rheumatoid Arthritis.<br>5. 18 years of age or over<br>6. Patient capable of giving informed consent<br>7. Willingness and ability to comply with scheduled visits, treatment plans and laboratory tests and other study procedures
Exclusion Criteria
1. Women who are pregnant or breast-feeding<br>2. Women of child-bearing potential, or males whose partners are women of child-bearing potential, unwilling to use effective contraception during the study and for at least 12 months after stopping study treatment.<br>3. History of or current primary inflammatory joint disease, or primary rheumatological autoimmune disease other than RA (if secondary to RA, then the patient is still eligible)<br>4. Prior exposure to Rituximab or Tocilizumab for the treatment of RA<br>5. Treatment with any investigational agent ≤ 4 weeks prior to baseline (or < 5 half-lives of the investigational drug, whichever is the longer).<br>6. Intra articular or parenteral corticosteroids ≤ 4 weeks prior to biopsy visit.<br>7. Active infection.<br>8. Septic arthritis within a native joint within the last 12 months.<br>9.Sepsis of a prosthetic joint within 12 months or indefinitely if the joint remains in situ.<br>10.Known HIV or active hepatitis B/C infection. Hepatitis B screening test must be performed at or in the preceding 3 months of screening visit.<br>11.Latent TB infection unless they have completed adequate antibiotic prophylaxis.<br>12.Malignancy (other than basal cell carcinoma) within the last 10 years<br>13.New York Heart Association (NYHA) grade 3 or 4 congestive cardiac failure.<br>14.Demyelinating disease.<br>15.Latex allergy or allergy to any excipients of Rituximab or Tocilizumab<br>16.Any other contra-indication to the study medications as detailed in their summaries of product characteristics (SmPC), including low IgG levels at clinician’s discretion.<br>17.Receipt of live vaccine <4 weeks prior to first infusion<br>18.Major surgery in 3 months prior to first infusion<br>19.Presence of a transplanted organ (with the exception of a corneal transplant >3 months prior to screening)<br>20.Known recent substance abuse (drug or alcohol)<br>21. Poor tolerability of venepuncture or lack of adequate venous access for required blood sampling during the study period.<br>22. Patients unable to tolerate synovial biopsy or in whom this is contraindicated including patients on anti-coagulants (oral anti-platelet agents are permitted)<br>23. Patients currently recruited to other clinical trial(s) involving an investigational medicinal product (except any observational follow-up periods not involving an IMP).<br>24. Other severe acute or chronic medical or psychiatric condition, or laboratory abnormality that would impart, in the judgment of the investigator, excess risk associated with study participation or study drug administration, or which, in the judgment of the investigator, would make the patient inappropriate for entry into this study.

Table 2: Additional secondary and supplementary endpoint analyses up to week 96

	Endpoints
1.	Patients deemed treatment failures at 16 weeks, will be switched to the other therapeutic option. Such patients will be considered a new patient starting at week 0 with treatment response assessed again at 16 weeks for primary response. Treatment difference before and after switch will be compared in B cell poor and B cell rich.
2.	For the B-cell rich synovial pathotypes, we aim to show non-inferiority of Rituximab compared to Tocilizumab. The same analysis as for the primary endpoint will be repeated.
	For the following endpoints, the treatment difference will be assessed separately in B cell poor, B cell rich and in the switches:
3.	Area under the curve (AUC) of mean improvement in DAS28 over time between 0 and16 weeks and between 0 and 48 weeks
4.	Percentage of patients with low disease activity (DAS28 < 3.2) at 16, 24, 36, 48, 96 weeks
5.	Percentage of patients in remission (DAS28 < 2.6) at 16, 48 and 96 weeks
6.	Percentage of patients with a low clinical disease activity index score (CDAI) at 16, 48 and 96 weeks
7.	Mean % change in DAS28 between baseline and 16, 48 and 96 weeks
8.	Mean % change in SF-36 score between baseline and 16, 48 and 96 weeks
9.	Mean % change in clinical disease activity index score (CDAI) between baseline and 16, 48 and 96 weeks
10.	Mean change in HAQ score between baseline and 16, 48 and 96 weeks
11.	Change in Fatigue score between baseline and 16, 48 and 96 weeks
12.	Serious adverse events over 12 months; the rate of serious adverse events in the 16 week period following a switch from one technology to the other will be compared
13.	Mean change in erosive score by the van der Heijde/Sharp scoring system at baseline and week 24
14.	Reduction in US 2D grey scale and power Doppler signal at Baseline, 16 and 48weeks.
15.	Mean change in synovial immune cell infiltrate determined immunohistologically (C20, CD68, CD138, CD3) between baseline, 16 and 48 weeks
16.	Mean change in synovial gene expression between baseline, 16 weeks and 48 weeks EULAR response based on DAS28 (good and moderate responder/non-responders)

Table 3: Sites and recruitment

Site Name	Principal Investigator	Randomised Patients
Bart's Health NHS Trust, London	Dr Fran Humby	62
Louvain, Belgium	Prof Patrick Durez	25
Lisbon, Portugal	Prof João Eurico Fonseca	9
Novara, Italy	Dr Pier Paolo Sainaghi	8
University Hospital of Wales, Cardiff	Prof Ernest Choy	6
Royal Victoria Infirmary, Newcastle	Prof John Isaacs	6
Southampton General Hospital	Prof Christopher Edwards	6
Basildon University Hospital	Dr Nagui Gendi	6
Barcelona, Spain	Dr Juan D Cañete	6
Southend University Hospital	Prof Bhaskar Dasgupta	4
Chapel Allerton Hospital, Leeds	Prof Maya Buch	4
Cagliari, Italy	Prof Alberto Cauli	4
Homerton University Hospital	Dr Piero Reynolds	3
Nuffield Orthopaedic Hospital, Oxford	Prof Peter Taylor	3
Aintree University Hospital, Liverpool	Prof Robert Moots	3
Manchester Royal Infirmary	Dr Pauline Ho	3
Guy's Hospital, London	Dr Nora Ng	3
Pavia, Italy	Prof Carlomaurizio Montecucco	2
Leuven, Belgium	Prof Patrick Verschueren	1

Table 4: Additional baseline patient demographics, stratified by IMP and histological classification

	Overall (n=161)				B cell poor (n=79)				B cell rich (n=64)			
	All patients (n=161)	Rituximab (n=82)	Tocilizumab (n=79)	p-value	All patients (n=79)	Rituximab (n=38)	Tocilizumab (n=41)	p-value	All patients (n=64)	Rituximab (n=33)	Tocilizumab (n=31)	p-value
Creatinine (μmol/L)	61 [53, 70]	63 [53, 73]	59 [54, 67]	0·44	61 [54, 71]	64 [54, 74]	59 [54, 67]	0·21	58 [52, 67]	59 [52, 67]	57 [50, 66]	0·62
Alanine aminotransferase (ALT), U/L	16 [12, 22]	16 [12, 21]	16 [12, 23]	0·66	16 [12, 22]	16 [13, 23]	16 [12, 20]	0·78	15 [10, 21]	15 [9, 19]	17 [12, 24]	0·17
Aspartate aminotransferase (AST), U/L	19 [15, 22]	19 [15, 22]	18 [16, 22]	0·91	19 [15, 22]	20 [15, 22]	19 [15, 22]	0·92	17 [15, 22]	17 [13, 22]	17 [16, 20]	0·50
Haemoglobin, g/L	123 [110, 131]	121 [109, 131]	123 [111, 131]	0·58	123 [110, 129]	123 [109, 129]	123 [111, 129]	0·99	120 [109, 131]	120 [108, 132]	119 [110, 130]	0·85
White Blood Cell count, 10^9/L	8·1 [6·8, 10·5]	8·00 [6·6, 10·2]	8·45 [7·0, 10·8]	0·41	7·90 [6·4, 9·9]	7·90 [6·8, 9·4]	8·10 [6·4, 10·1]	0·98	8·9 [7·2, 10·9]	8·35 [7·1, 10·7]	9·33 [7·5, 11·8]	0·27
Platelets, 10^9/L	303 [254, 384]	302 [256, 344]	304 [251, 394]	0·51	291 [242, 383]	292 [255, 335]	283 [234, 393]	0·83	314 [256, 408]	308 [255, 374]	339 [257, 441]	0·34
Neutrophils, 10^9/L	5·70 [4·43, 7·22]	5·70 [4·20, 7·30]	5·60 [4·62, 7·11]	0·66	5·44 [4·20, 6·90]	5·69 [4·23, 6·98]	5·40 [3·97, 6·90]	0·55	6·20 [4·56, 7·77]	6·10 [4·22, 7·73]	6·45 [4·88, 8·42]	0·27
Lymphocytes, 10^9/L	1·70 [1·33, 2·30]	1·70 [1·20, 2·30]	1·80 [1·40, 2·40]	0·15	1·67 [1·20, 2·25]	1·51 [1·13, 2·19]	1·80 [1·37, 2·47]	0·17	1·77 [1·32, 2·38]	1·79 [1·30, 2·34]	1·73 [1·41, 2·38]	0·71
Patient's global assessment—arthritis, 0–100 VAS	72 [51, 85]	71 [50, 82]	74 [51, 87]	0·37	72 [49, 82]	72 [49, 81]	71 [51, 85]	0·80	71 [48, 89]	64 [39, 79]	77 [53, 90]	0·12
Physician's global assessment, 0–100 VAS	61 [48, 78]	60 [49, 79]	64 [46, 76]	0·87	60 [49, 76]	60 [49, 76]	63 [49, 73]	0·77	60 [44, 80]	58 [46, 80]	66 [40, 76]	0·89
Patient's assessment of early morning stiffness, 0–100 VAS	45 [20, 100]	35 [20, 100]	60 [20, 100]	0·45	30 [16, 90]	30 [16, 82]	45 [18, 92]	0·55	60 [20, 100]	60 [15, 100]	60 [30, 100]	0·36
Patient's assessment of tiredness, 0–100 VAS	68 [50, 83]	67 [44, 78]	70 [50, 86]	0·15	69 [50, 81]	68 [36, 76]	70 [51, 86]	0·40	63 [44, 83]	54 [36, 79]	75 [49, 89]	0·082
Patient's assessment of pain, 0–100 VAS	70 [45, 86]	66 [48, 83]	72 [43, 87]	0·50	68 [42, 81]	66 [45, 79]	71 [41, 85]	0·41	67 [43, 91]	65 [42, 89]	72 [48, 91]	0·34
HAQ total score	1·75 [1·25, 2·13]	1·75 [1·25, 2·13]	1·75 [1·25, 2·13]	0·97	1·81 [1·25, 2·25]	1·75 [1·25, 2·00]	1·88 [1·38, 2·27]	0·50	1·63 [1·25, 2·13]	1·63 [1·13, 2·13]	1·75 [1·38, 2·13]	0·65
Functional Assessment of Chronic Illness Therapy (FACIT) score	22 [14, 32]	23 [15, 32]	21 [13, 33]	0·56	22 [15, 32]	23 [15, 32]	21 [13, 33]	0·66	22 [14, 32]	25 [16, 32]	19 [13, 32]	0·15
Short Form-36												
Physical functioning, 0-100	30 [15, 45]	30 [10, 48]	30 [15, 45]	0·94	25 [15, 40]	25 [11, 40]	25 [15, 40]	0·85	32 [15, 51]	35 [15, 55]	30 [17, 47]	0·71
Physical role functioning, 0-100	0 [0, 25]	0 [0, 25]	0 [0, 0]	0·33	0 [0, 25]	0 [0, 25]	0 [0, 12]	0·77	0 [0, 25]	0 [0, 50]	0 [0, 0]	0·13
Emotional role functioning, 0-100	0 [0, 66]	33 [0, 91]	0 [0, 66]	0·31	0 [0, 75]	33 [0, 91]	0 [0, 66]	0·31	0 [0, 66]	0 [0, 66]	0 [0, 50]	0·49
Vitality, 0-100	35 [20, 45]	35 [20, 50]	30 [20, 45]	0·42	34 [20, 50]	34 [20, 50]	32 [20, 45]	0·60	35 [15, 46]	40 [20, 50]	30 [15, 40]	0·16
Mental health, 0-100	60 (19)	61 (19)	58 (20)	0·43	59 (19)	61 (19)	58 (20)	0·57	60 (19)	64 (17)	56 (21)	0·10

Social role functioning, 0-100	37 [25, 62]	37 [25, 62]	50 [25, 75]	0·45	50 [25, 62]	50 [25, 62]	50 [25, 62]	0·74	37 [25, 62]	37 [25, 50]	50 [25, 75]	0·43
Bodily pain, 0-100	22 [12, 45]	22 [22, 45]	22 [10, 45]	0·35	22 [22, 45]	22 [22, 44]	22 [10, 45]	0·28	22 [12, 45]	22 [20, 45]	22 [10, 45]	0·49
General health perceptions, 0-100	35 [25, 50]	35 [25, 45]	35 [25, 50]	0·67	32 [25, 50]	35 [25, 48]	27 [23, 50]	0·73	35 [25, 50]	35 [25, 45]	40 [35, 50]	0·16
Previous Methotrexate use	161 (100%)	82 (100%)	79 (100%)	··	79 (100%)	38 (100%)	41 (100%)	··	64 (100%)	33 (100%)	31 (100%)	··
Previous Prednisolone use	90 (56%)	44 (54%)	46 (58%)	0·67	42 (53%)	23 (61%)	19 (46%)	0·30	40 (62%)	18 (55%)	22 (71%)	0·27
Number of concomitant DMARDs				0·51				0·75				0·73
0	85 (53%)	42 (51%)	43 (54%)		44 (56%)	19 (50%)	25 (61%)		34 (53%)	18 (55%)	16 (52%)	
1	31 (19%)	14 (17%)	17 (22%)		16 (20%)	8 (21%)	8 (20%)		12 (19%)	5 (15%)	7 (23%)	
2	32 (20%)	20 (24%)	12 (15%)		14 (18%)	8 (21%)	6 (15%)		13 (20%)	8 (24%)	5 (16%)	
3+	13 (8%)	6 (7%)	7 (9%)		5 (6%)	3 (8%)	2 (5%)		5 (8%)	2 (6%)	3 (10%)	

Data are n (%), median [IQR] or mean (SD), HAQ=health assessment questionnaire, VAS=visual analogue scale.

Table 5: Clinical outcomes at 16 weeks, B cell poor population (histological classification), per-protocol (PP) analysis set

Variable[1]	Rituximab (n=37)	Tocilizumab (n=30)	Treatment effect	Unadjusted p value
Primary endpoint	n (%)	n (%)	Risk difference (95% CIs)	
CDAI ≥50% improvement at week 16	16 (43·2)	21 (70)	26·8% (3·9% to 49·6%)	0·029
Supplementary endpoint				
CDAI ≥50% improvement and CDAI ≤10.1 at week 16	9 (24·3)	17 (56·7)	32·3% (9·9% to 54·8%)	0·0069
Binary secondary endpoints				
CDAI ≤10.1 at week 16	11 (29·7)	17 (56·7)	26·9% (3·9% to 50·0%)	0·026
DAS28 (ESR) ≤3.2 at week 16	10 (27·0)	14 (46·7)	19·6% (-3·2% to 42·5%)	0·095
DAS28 (CRP) ≤3.2 at week 16	12 (32·4)	16 (53·3)	20·9% (-2·5% to 44·3%)	0·085
DAS28 (ESR) ≤2.6 at week 16	6 (16·2)	13 (43·3)	27·1% (5·8% to 48·5%)	0·014
DAS28 (CRP) ≤2.6 at week 16	7 (18·9)	11 (36·7)	17·7% (-3·6% to 39·1%)	0·10
Moderate/Good EULAR DAS28(ESR) response at week 16	24 (64·9)	29 (96·7)	31·8% (15·1% to 48·5%)	0·0018
Moderate/Good EULAR DAS28(CRP) response at week 16	21 (56·8)	27 (90·0)	33·2% (14·0% to 52·5%)	0·0029
Continuous secondary endpoints			Least squares mean difference (95% CIs)	
CDAI, least squares mean change at week 16	-11·93 (1·92)	-17·88 (2·13)	5·95 (0·21 to 11·7)	0·043
DAS28(ESR), least squares mean change at week 16	-1·47 (0·21)	-2·85 (0·24)	1·38 (0·74 to 2·02)	<0·0001
DAS28(CRP), least squares mean change at week 16	-1·3 (0·21)	-2·2 (0·23)	0·90 (0·27 to 1·52)	0·0054
HAQ, least squares mean change at week 16	-0·25 (0·08)	-0·4 (0·09)	0·14 (-0·11 to 0·39)	0·26
FACIT, least squares mean change at week 16	1·75 (1·10)	5·56 (1·18)	-3·81 (-7·04 to -0·58)	0·021
SF36 - PCS, least squares mean change at week 16	4·25 (1·50)	8·25 (1·67)	-4·00 (-8·49 to 0·49)	0·08
SF36 - MCS, least squares mean change at week 16	-0·69 (1·57)	2·65 (1·75)	-3·35 (-8·07 to 1·37)	0·16

[1]Data is here expressed as n (%) for primary, supplementary and binary secondary endpoints and as least squares mean (SD) for continuous secondary endpoints. CDAI: Clinical Disease Activity Index; DAS28: 28 joint count Disease Activity Score; EULAR: European League against Rheumatism; CRP=C-reactive protein. ESR=erythrocyte sedimentation rate; SD: Standard Deviation; HAQ=Health Assessment Questionnaire. SF36-PCS=Physical Components Summary of the SF-36 questionnaire. SF36-MCS=Mental Components Summary of the SF-36 questionnaire.
.

Table 6: Clinical outcomes at 16 weeks, B cell poor population (RNA-seq classification), per-protocol (PP) analysis set

Variable[1]	Rituximab (n=32)	Tocilizumab (n=25)	Treatment effect	Unadjusted p value
Primary endpoint	n (%)	n (%)	Percentage difference (95% CIs)	
CDAI ≥50% improvement at week 16	11 (34·4)	19 (76)	41·6% (18·1% to 65·1%)	0·0018
Supplementary endpoint				
CDAI ≥50% improvement and CDAI ≤10.1 at week 16	4 (12·5)	15 (60)	47·5% (25·1% to 69·9%)	0·00022
Binary secondary endpoints				
CDAI ≤10.1 at week 16	5 (15·6)	15 (60·0)	44·4% (21·4% to 67·3%)	0·0007
DAS28 (ESR) ≤3.2 at week 16	6 (18·8)	15 (60·0)	41·2% (17·8% to 64·7%)	0·0014
DAS28 (CRP) ≤3.2 at week 16	7 (21·9)	15 (60·0)	38·1% (14·2% to 62·1%)	0·0033
DAS28 (ESR) ≤2.6 at week 16	3 (9·4)	12 (48·0)	38·6% (16·6% to 60·7%)	0·0019
DAS28 (CRP) ≤2.6 at week 16	4 (12·5)	9 (36·0)	23·5% (1·5% to 45·5%)	0·056
Moderate/Good EULAR DAS28(ESR) response at week 16	20 (62·5)	25 (100·0)	37·5% (20·7% to 54·3%)	0·0005
Moderate/Good EULAR DAS28(CRP) response at week 16	17 (53·1)	23 (92·0)	38·9% (18·6% to 59·2%)	0·0015
Continuous secondary endpoints			Least squares mean difference (95% CIs)	
CDAI, least squares mean change at week 16	-10·72 (1·95)	-18·63 (2·21)	7·91 (1·99 to 13·83)	0·0097
DAS28(ESR), least squares mean change at week 16	-1·27 (0·22)	-2·99 (0·25)	1·72 (1·05 to 2·40)	<0·0001
DAS28(CRP), least squares mean change at week 16	-1·11 (0·21)	-2·34 (0·24)	1·22 (0·58 to 1·86)	0·0003
HAQ, least squares mean change at week 16	-0·21 (0·08)	-0·23 (0·09)	0·02 (-0·23 to 0·27)	0·86
FACIT, least squares mean change at week 16	2·32 (1·43)	4·24 (1·60)	-1·92 (-6·23 to 2·38)	0·37
SF36 - PCS, least squares mean change at week 16	3·73 (1·51)	4·22 (1·71)	-0·49 (-5·07 to 4·09)	0·83
SF36 - MCS, least squares mean change at week 16	0·99 (1·81)	5·12 (2·06)	-4·13 (-9·63 to 1·37)	0·14

[1] Data is here expressed as n (%) for primary, supplementary and binary secondary endpoints and as least squares mean (SD) for continuous secondary endpoints. CDAI: Clinical Disease Activity Index; DAS28: 28 joint count Disease Activity Score; EULAR: European League against Rheumatism; CRP=C-reactive protein. ESR=erythrocyte sedimentation rate; SD: Standard Deviation; HAQ=Health Assessment Questionnaire. SF36-PCS=Physical Components Summary of the SF-36 questionnaire. SF36-MCS=Mental Components Summary of the SF-36 questionnaire.

Table 7: Clinical outcomes at 16 weeks, B cell rich population (histological classification), per-protocol (PP) analysis set

Variable[1]	Rituximab (n=31)	Tocilizumab (n=23)	Treatment effect	Unadjusted p value
Primary endpoint	n (%)	n (%)	Percentage difference (95% CIs)	
CDAI ≥50% improvement at week 16	12 (38·7)	12 (52·2)	13·5% (-13·2% to 40·1%)	0·32
Supplementary endpoint				
CDAI ≥50% improvement and CDAI ≤10.1 at week 16	5 (16·1)	7 (30·4)	14·3% (-8·5% to 37·1%)	0·32
Binary secondary endpoints				
CDAI ≤10.1 at week 16	7 (22·6)	8 (34·8)	12·2% (-12·2% to 36·6%)	0·32
DAS28 (ESR) ≤3.2 at week 16	8 (25·8)	11 (47·8)	22·0% (-3·6% to 47·6%)	0·094
DAS28 (CRP) ≤3.2 at week 16	10 (32·3)	10 (43·5)	11·2% (-14·9% to 37·3%)	0·40
DAS28 (ESR) ≤2.6 at week 16	2 (6·5)	9 (39·1)	32·7% (10·9% to 54·4%)	0·0052
DAS28 (CRP) ≤2.6 at week 16	4 (12·9)	7 (30·4)	17·5% (-4·7% to 39·7%)	0·17
Moderate/Good EULAR DAS28(ESR) response at week 16	23 (74·2)	21 (91·3)	17·1% (-2·1% to 36·3%)	0·16
Moderate/Good EULAR DAS28(CRP) response at week 16	21 (67·7)	18 (78·3)	10·5% (-13·0% to 34·1%)	0·54
Continuous secondary endpoints			Least squares mean difference (95% CIs)	
CDAI, least squares mean change at week 16	-12·84 (2·1)	-12·58 (2·43)	-0·27 (-6·72 to 6·19)	0·93
DAS28(ESR), least squares mean change at week 16	-1·42 (0·22)	-2·62 (0·26)	1·20 (0·51 to 1·89)	0·0009
DAS28(CRP), least squares mean change at week 16	-1·39 (0·21)	-1·97 (0·24)	0·58 (-0·06 to 1·22)	0·076
HAQ, least squares mean change at week 16	-0·28 (0·09)	-0·3 (0·10)	0·02 (-0·24 to 0·28)	0·88
FACIT, least squares mean change at week 16	7·76 (1·91)	7·45 (2·19)	0·31 (-5·55 to 6·18)	0·92
SF36 - PCS, least squares mean change at week 16	6·35 (1·83)	6·41 (2·09)	-0·07 (-5·66 to 5·52)	0·98
SF36 - MCS, least squares mean change at week 16	4·37 (2·25)	4·18 (2·58)	0·19 (-6·70 to 7·08)	0·96

[1]Data is here expressed as n (%) for primary, supplementary and binary secondary endpoints and as least squares mean (SD) for continuous secondary endpoints. CDAI: Clinical Disease Activity Index; DAS28: 28 joint count Disease Activity Score; EULAR: European League against Rheumatism; CRP=C-reactive protein. ESR=erythrocyte sedimentation rate; SD: Standard Deviation; HAQ=Health Assessment Questionnaire. SF36-PCS=Physical Components Summary of the SF-36 questionnaire. SF36-MCS=Mental Components Summary of the SF-36 questionnaire.

Table 8: Clinical outcomes at 16 weeks, B cell rich population (RNA-seq classification), per-protocol (PP) analysis set

Variable[1]	Rituximab (n=28)	Tocilizumab (n=19)	Treatment effect	Unadjusted p value
Primary endpoint	n (%)	n (%)	Percentage difference (95% CIs)	
CDAI ≥50% improvement at week 16	14 (50)	9 (47·4)	-2·6% (-31·7% to 26·5%)	0·86
Supplementary endpoint				
CDAI ≥50% improvement and CDAI ≤10.1 at week 16	7 (25)	4 (21·1)	-3·9% (-28·3% to 20·4%)	1
Binary secondary endpoints				
CDAI ≤10.1 at week 16	10 (35·7)	5 (26·3)	-9·4% (-36·0% to 17·2%)	0·54
DAS28 (ESR) ≤3.2 at week 16	9 (32·1)	7 (36·8)	4·7% (-23·0% to 32·4%)	0·74
DAS28 (CRP) ≤3.2 at week 16	12 (42·9)	7 (36·8)	-6·0% (-34·4% to 22·4%)	0·68
DAS28 (ESR) ≤2.6 at week 16	3 (10·7)	7 (36·8)	26·1% (1·6% to 50·7%)	0·066
DAS28 (CRP) ≤2.6 at week 16	4 (14·3)	5 (26·3)	12·0% (-11·6% to 35·7%)	0·45
Moderate/Good EULAR DAS28(ESR) response at week 16	22 (78·6)	17 (89·5)	10·9% (-9·6% to 31·4%)	0·44
Moderate/Good EULAR DAS28(CRP) response at week 16	21 (75·0)	15 (78·9)	3·9% (-20·4% to 28·3%)	1·00
Continuous secondary endpoints			Least squares mean difference (95% CIs)	
CDAI, least squares mean change at week 16	-14·03 (2·18)	-12·1 (2·64)	-1·92 (-8·83 to 4·99)	0·58
DAS28(ESR), least squares mean change at week 16	-1·66 (0·22)	-2·44 (0·27)	0·78 (0·08 to 1·47)	0·03
DAS28(CRP), least squares mean change at week 16	-1·57 (0·21)	-1·81 (0·26)	0·24 (-0·43 to 0·91)	0·47
HAQ, least squares mean change at week 16	-0·27 (0·08)	-0·42 (0·10)	0·15 (-0·11 to 0·41)	0·24
FACIT, least squares mean change at week 16	6·02 (1·71)	8·13 (2·04)	-2·11 (-7·51 to 3·29)	0·44
SF36 - PCS, least squares mean change at week 16	6·18 (1·76)	8·87 (2·10)	-2·68 (-8·20 to 2·84)	0·33
SF36 - MCS, least squares mean change at week 16	3·12 (2·31)	4·16 (2·76)	-1·04 (-8·36 to 6·29)	0·78

[1]Data is here expressed as n (%) for primary, supplementary and binary secondary endpoints and as least squares mean (SD) for continuous secondary endpoints. CDAI: Clinical Disease Activity Index; DAS28: 28 joint count Disease Activity Score; EULAR: European League against Rheumatism; CRP=C-reactive protein. ESR=erythrocyte sedimentation rate; SD: Standard Deviation; HAQ=Health Assessment Questionnaire. SF36-PCS=Physical Components Summary of the SF-36 questionnaire. SF36-MCS=Mental Components Summary of the SF-36 questionnaire.

Table 9: CDAI 50% response rates according to seropositivity for RF and/or ACPA

	Rituximab (n=82)		Tocilizumab(n=79)		
	Responders (n=37)	Non Responders (n=45)	Responders (n=44)	Non Responders (n=35)	p-value
Rheumatoid factor (RF) status					
Positive	28 (75·7%)	36 (80·0%)	30 (68·2%)	25 (71·4%)	0·24
Negative	9 (24·3%)	9 (20·0%)	14 (31·8%)	10 (28·6%)	0·76
Anti–citrullinated protein antibody (ACPA) status					
Positive	30 (81·1%)	37 (82·2%)	37 (84·1%)	24 (68·6%)	0·072
Negative	7 (18·9%)	8 (17·8%)	7 (15·9%)	11 (31·4%)	0·73
Rheumatoid factor (RF) OR Anti–citrullinated protein antibody (ACPA) status					
Positive	33 (89·2%)	40 (88·9%)	38 (86·4%)	29 (82·9%)	0·17
Negative	4 (10·8%)	5 (11·1%)	6 (13·7%)	6 (17·1%)	1

Table 10: Non-serious Adverse events from Week 0 to Week 48 (+30 days), safety analysis set (SAF)

Event[1]	Total (n=225)	Rituximab (n=108)	Tocilizumab (n=117)	Percentage difference (95% CIs)
Any non-serious adverse event	170 (75·6%)	76 (70·4%)	94 (80·3%)	10·0% (-1·3% to 21·2%)
Blood and lymphatic system disorders	9 (4·0%)	2 (1·9%)	7 (6·0%)	4·1% (-0·9% to 9·1%)
Cardiac disorders	1 (0·4%)	1 (0·9%)	0	
Ear and labyrinth disorders	5 (2·2%)	2 (1·9%)	3 (2·6%)	0·7% (-3·1% to 4·5%)
Epidermal and dermal conditions	1 (0·4%)	1 (0·9%)	0	
Eye disorders	9 (4·0%)	5 (4·6%)	4 (3·4%)	-1·2% (-6·4% to 3·9%)
Gastrointestinal disorders	49 (21·8%)	24 (22·2%)	25 (21·4%)	-0·9% (-11·7% to 9·9%)
General disorders and administration site conditions	34 (15·1%)	12 (11·1%)	22 (18·8%)	7·7% (-1·5% to 16·9%)
Hepatobiliary disorders	3 (1·3%)	2 (1·9%)	1 (0·9%)	-1·0% (-4·0% to 2·0%)
Immune system disorders	12 (5·3%)	7 (6·5%)	5 (4·3%)	-2·2% (-8·1% to 3·7%)
Infections and infestations	60 (26·7%)	28 (25·9%)	32 (27·4%)	1·4% (-10·1% to 13·0%)
Injury, poisoning and procedural complications	21 (9·3%)	8 (7·4%)	13 (11·1%)	3·7% (-3·8% to 11·2%)
Investigations	29 (12·9%)	14 (13·0%)	15 (12·8%)	-0·1% (-8·9% to 8·6%)
Metabolism and nutrition disorders	2 (0·9%)	1 (0·9%)	1 (0·9%)	-0·1% (-2·5% to 2·4%)
Musculoskeletal and connective tissue disorders	36 (16·0%)	18 (16·7%)	18 (15·4%)	-1·3% (-10·9% to 8·3%)
Neoplasms benign, malignant and unspecified (incl cysts and polyps)	1 (0·4%)	0	1 (0·9%)	
Nervous system disorders	21 (9·3%)	12 (11·1%)	9 (7·7%)	-3·4% (-11·1% to 4·2%)
Psychiatric disorders	6 (2·7%)	2 (1·9%)	4 (3·4%)	1·6% (-2·6% to 5·7%)
Renal and urinary disorders	26 (11·6%)	8 (7·4%)	18 (15·4%)	8·0% (-0·2% to 16·2%)
Reproductive system and breast disorders	4 (1·8%)	2 (1·9%)	2 (1·7%)	-0·1% (-3·6% to 3·3%)
Respiratory, thoracic and mediastinal disorders	69 (30·7%)	32 (29·6%)	37 (31·6%)	2·0% (-10·1% to 14·0%)
Skin and subcutaneous tissue disorders	46 (20·4%)	21 (19·4%)	25 (21·4%)	1·9% (-8·6% to 12·5%)
Surgical and medical procedures	13 (5·8%)	4 (3·7%)	9 (7·7%)	4·0% (-2·0% to 10·0%)
Vascular disorders	26 (11·6%)	14 (13·0%)	12 (10·3%)	-2·7% (-11·1% to 5·7%)

[1] Data is here expressed as N (%) and include all events reported after the first prescription of IMP and up to Week 48 + 30 days. Some patients had more than one adverse event. Events are classified using the Medical Dictionary for Regulatory Activities (MedDRA) system classification, using the System Organ Classes (SOCs) grouping.

Table 11: Summary of Major Protocol Violations

#	Description of Protocol Violation	# of occurrences
1	Patient missed tocilizumab infusion prior to week 16	5
2	Steroid injection given <4 weeks prior to baseline visit	2
3	Patient commenced corticosteroid <4 weeks prior to baseline	2
4	Patient had previously been diagnosed with melanoma (exclusion criteria)	1
5	VAS Pain Score not completed at week 16 (CDAI could not be calculated)	1
6	Patient did not attend week 16 visit	5
7	Time between visits was outside the acceptable window	4
8	Patient ceased treatment prior to week 16	5
**Total**		**25**

Histological analysis: Legend to supplementary figures
A minimum of 6 synovial biopsies were paraffin embedded en masse and sections stained for Hematoxylin and Eosin (H&E), and immune-histochemical markers CD20 (B cells), CD3 (T cells), CD138 (plasma cells) and CD68 (macrophages) as previously described (3,19). Sections underwent semi-quantitative scoring (0-4) to determine expression of CD20+ B cells, CD3+ T cells, CD138+ plasma cells and CD68+ lining (l) and sub lining (sl) macrophages (figure 1) adapted from a previously described score (19,20). H&E stained slides also underwent evaluation to determine the level of synovitis (21). If CD20+ve cells were identified staining for CD21 (follicular dendritic cells, FDC) was also performed as previously described. (3) Patients were classified as B-cell rich or B-cell poor in the NHS pathology laboratory of Barts Health NHS Trust by a consultant pathologist (HR) followed by an independent histological classification in the rheumatology research laboratories at QMUL by a second expert in synovial pathology (GT), according to a validated algorithm shown in figure 2. Synovial tissue with a CD20 score ≥2 and with CD20+ B cell aggregates were classified as B cell rich as previously described (19). Synovial tissue with CD20 score <2 were classified as B cell poor (19). Any discrepancies in classification were resolved through mutual agreement. Patients in which definite synovial tissue could not be identified were classified as “unknown”. B-cell rich samples were further classified as germinal centre (GC)+ve if CD21+ FDC networks were subsequently identified (figure 3). As predefined in the study protocol only patients classified as B-cell rich or B-cell poor were included in the primary analysis of the trial with examination of the GC+ve cohort to be undertaken as part of a subsequent exploratory analysis.

Figure 1: Reference atlas for histological scores for synovial tissue (0-4)

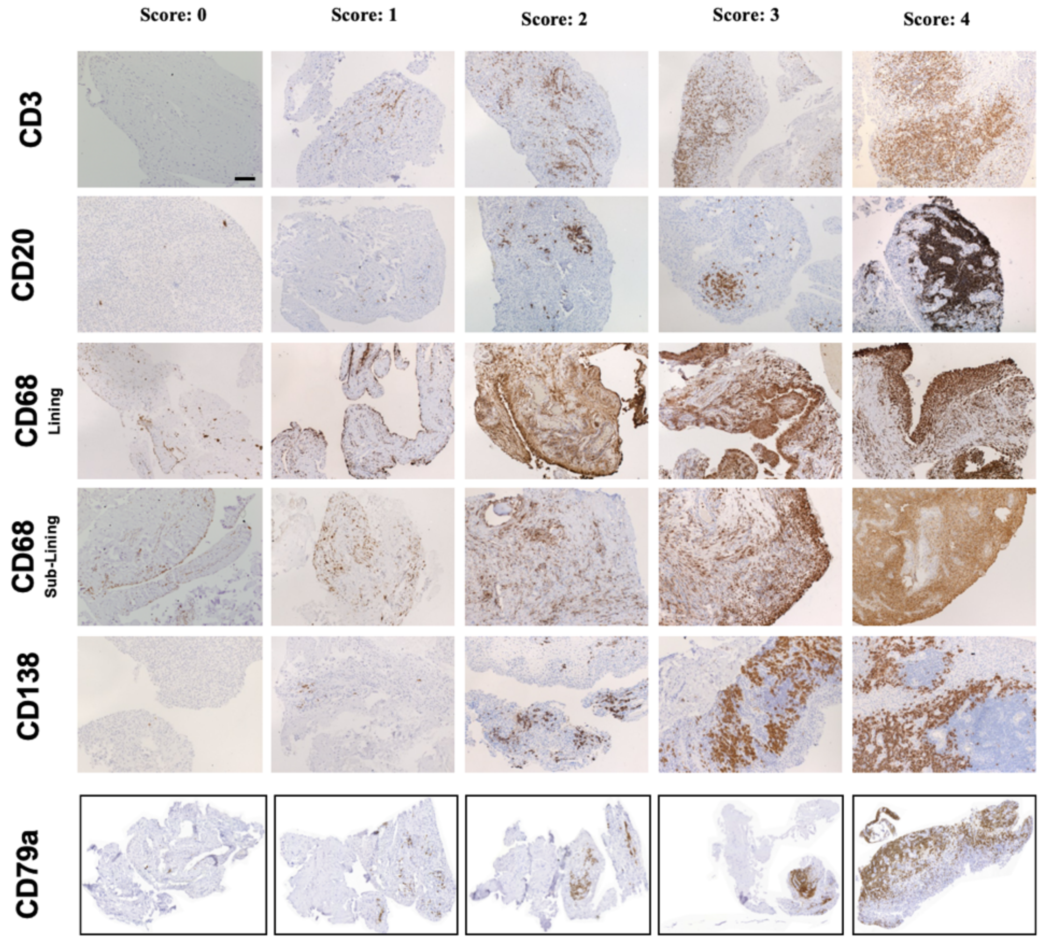

Figure 2: Histological classification of synovial tissue

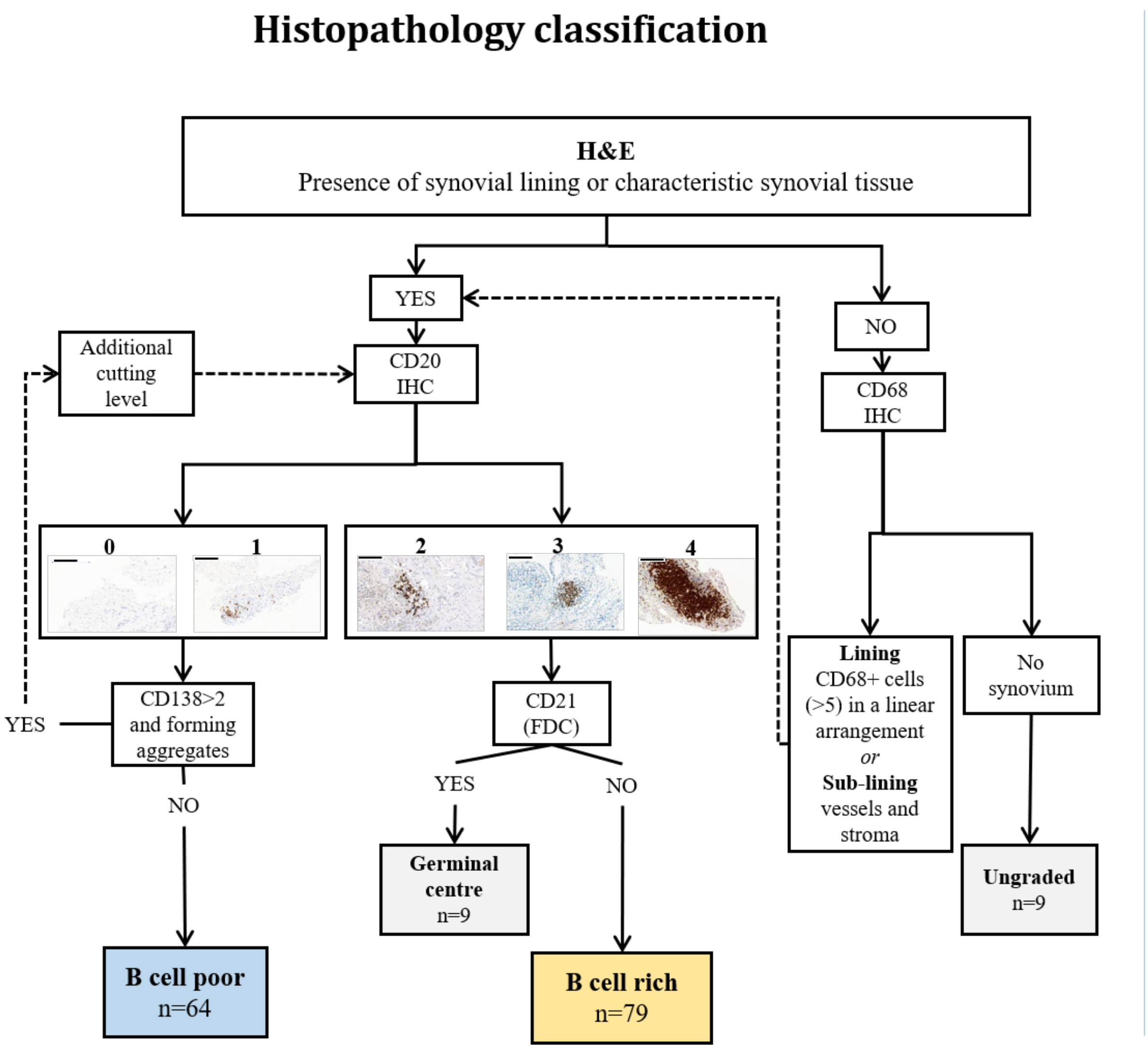

Figure 3: Reference atlas for immunohistochemical scores for CD21 stained synovial tissue

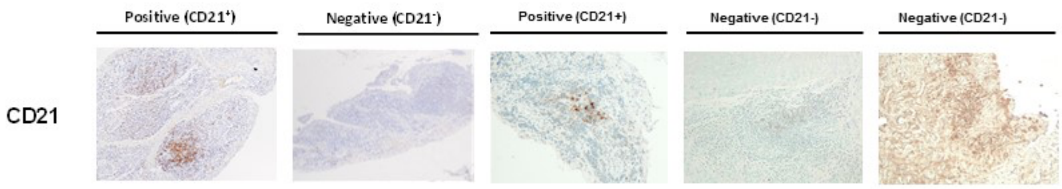

RNA-seq analysis: Legend to supplementary figure

Synovial tissue from 162 patients was available for RNA extraction and RNA-sequencing. A minimum of 6 synovial samples per patient were immediately immersed in RNA-Later and RNA extracted using either Phenol/Chloroform or via a Zymo Direct-zol™ RNA MicroPrep - Total RNA/miRNA Extraction kit as previously described (ref. 20). All RNA samples were transferred to Genewiz for RNA sequencing by Illumina HiSeq. 184 paired-end RNA-seq samples of 50 million reads of 150 base pairs were trimmed to remove the Illumina adaptors using bbduk from the BBMap package version 37.93 using the default parameters. Transcripts were then quantified using Salmon version 0.13.1(22) and an index generated from the Gencode release 29 transcriptome following the standard operating procedure. Tximport version 1.13.10 was used to aggregate the transcript level expression data to genes, counts were then subject to variance stabilising transform (VST) using the DESEQ2 version 1.25.9 package (ref. 24). Following exclusion of patients classified histologically as GC+ (n=9) 153 patients remained. One patient was withdrawn before IMP was administered and 28 were excluded following RNAseq quality control or due to poor mapping. Therefore, 124 patients had RNAseq data available for subsequent analysis. Patients were classified as B cell poor/rich according to a previously developed B cell-specific gene module derived from analysis of FANTOM5 gene expression data (ref. 25). As no pre-determined cut-off points for B cell transcript classification were found in the literature and to avoid potential bias, patients were classified as B cell poor/rich according to the median transcript module value as shown in figure 4.

Figure 4: Heat map of RNA-seq B cell module gene expression across whole cohort

Samples are ranked by RNA-seq B cell module score from lowest to highest demonstrating reclassification of patients into RNA-seq B cell poor and rich categories. Top tracks show original histology class, CD20 and CD138 histology scores. GC: germinal centre as classified by histology.

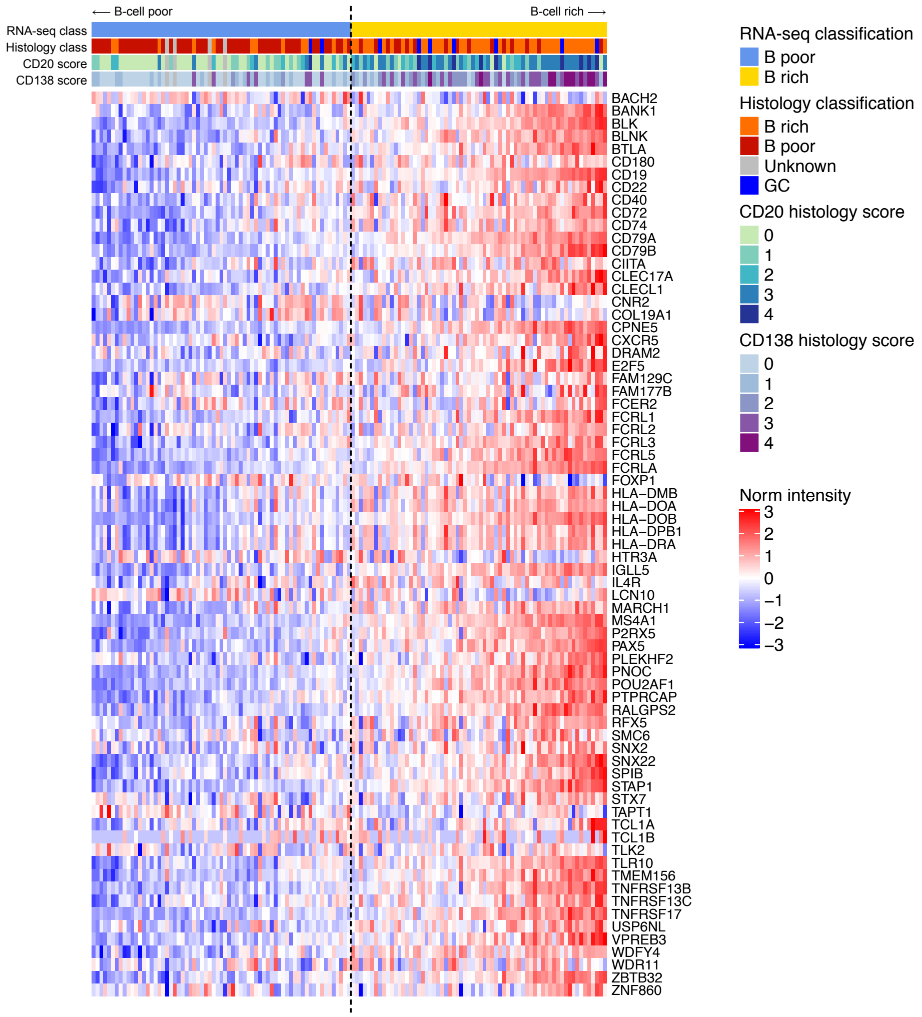

Figure 5: Testing the cut-off of the RNA-Seq B cell module for defining B cell poor/rich in R4RA

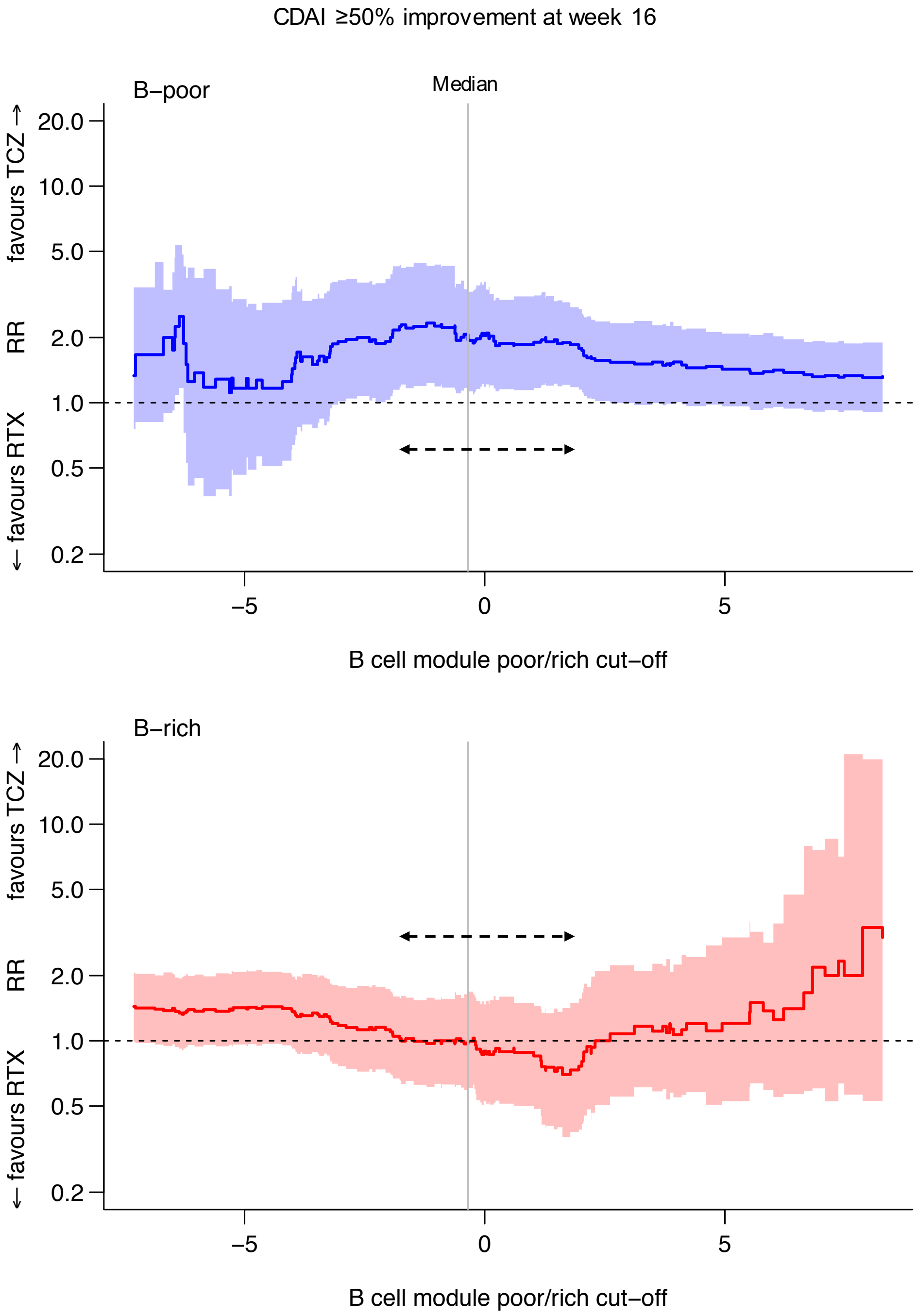

Plot showing how the risk ratio (y axis) for Tocilizumab (TCZ) vs Rituximab (RTX) for CDAI 50% responders at week 16 in B cell poor (blue, above) and B cell rich (red, below) groups varies if the RNA-seq B cell module cut-off point is varied (x axis). Vertical grey line shows median RNA-seq B module score corresponding to original analysis of study using median B cell score as cut-off. At the median, the lower bound for the 95% CI for B cell poor is significantly >1.0 confirming significant result for B cell poor, but not for B cell rich. Dashed horizontal lines show range over which statistical significance is maintained.

Figure 6: Schematic of clinical trial design

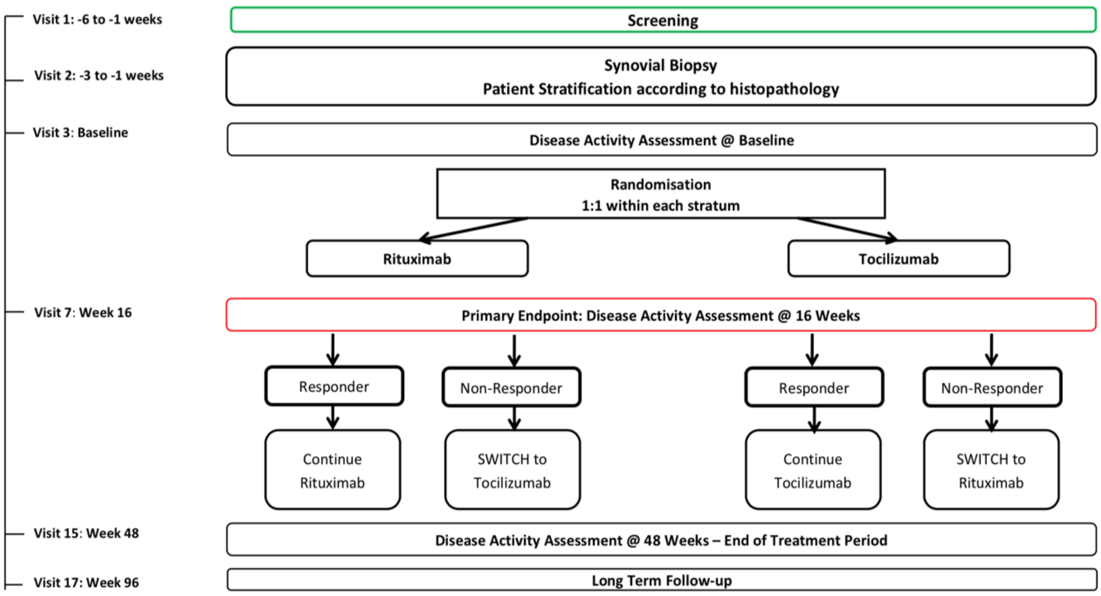

Supplement: Supplementary appendix [file mmc1.pdf]
